# Novel *M*. *tuberculosis* specific IL-2 ELISpot assay discriminates adult patients with active or latent tuberculosis

**DOI:** 10.1371/journal.pone.0197825

**Published:** 2018-06-01

**Authors:** Chiara Della Bella, Michele Spinicci, Alessia Grassi, Filippo Bartalesi, Marisa Benagiano, Katja Truthmann, Simona Tapinassi, Arianna Troilo, Sofia D’Elios, Heba Alnwaisri, Eduard Shuralev, Mahavir Singh, Alessandro Bartoloni, Mario Milco D’Elios

**Affiliations:** 1 Department of Experimental and Clinical Medicine, University of Florence, Florence, Italy; 2 Infectious and Tropical Diseases Unit, Florence Careggi University Hospital, Florence, Italy; 3 LIONEX Diagnostics and Therapeutics GmbH, Braunschweig, Germany; 4 Department of Clinical and Experimental Medicine, University of Pisa, Pisa, Italy; 5 Institute of Environmental Sciences, Kazan Federal University, and Central Research Laboratory, Kazan State Medical Academy, and Federal Center for Toxicological, Radiation and Biological Safety, Kazan, Tatarstan, Russian Federation; Universita degli Studi di Palermo, ITALY

## Abstract

**Background:**

Tuberculosis (TB) still is a major worldwide health problem, with 10.4 million new cases in 2016. Only 5–15% of people infected with *M*. *tuberculosis* develop TB disease while others remain latently infected (LTBI) during their lifetime. Thus, the absence of tests able to distinguish between latent infection and active tuberculosis is one of the major limits of currently available diagnostic tools.

**Methods:**

A total of 215 patients were included in the study as active TB cases (n = 73), LTBI subjects (n = 88) and healthy persons (n = 54). Peripheral blood mononuclear cells (PBMCs) were isolated from each patient and the LIOSpot® TB anti-human IL-2 ELISpot assay was performed to test their proliferative response to *M*. *tuberculosis* antigens ESAT-6, CFP-10 and Ala-DH. Statistical analysis was performed to define the sensitivity and the specificity of the LIOSpot® TB kit for each antigen used and to set the best cut off value that enables discrimination between subjects with active TB or latent TB infection.

**Results:**

Comparing the LIOSpot® TB results for each tested antigen between uninfected and infected subjects and between people with latent infection and active TB disease, the differences were significant for each antigen (p< 0.0001) but the ROC analysis demonstrated a high accuracy for the Ala-DH test only, with a cut off value of 12.5 SFC per million PBMCs and the Ala-DH ROC curve conferred a 96% sensitivity and 100% specificity to the test. For the ESAT-6 antigen, with a best cut off value of 71.25 SFC per million PBMCs, a sensitivity of 86% and specificity of 36% was obtained. Finally, the best cut off value for CFP-10 was 231.25 SFC per million PBMCs, with a sensitivity of 80% and a specificity of 54%. Thus, as for IGRA assays such as Quantiferon and T-Spot TB tests, ESAT-6 and CFP-10 are unable to distinguish LTBI from active TB when IL-2 is measured. On the contrary, the IL-2 production induced by Ala-DH, measured by LIOSpot® TB kit, shows high sensitivity and specificity for active TB disease.

**Conclusions:**

This study demonstrates that the LIOSpot® TB test is a highly useful diagnostic tool to discriminate between latent TB infection and active tuberculosis in adults patients.

## Introduction

Tuberculosis (TB) still is a major health problem worldwide. In 2016, there were 10.4 million new estimated cases worldwide. TB ranks above HIV/AIDS as one of the leading causes of death from an infectious disease; 1.7 million deaths were estimated in 2016 [1].

Incident TB cases reflect a little percentage of the global infection burden: indeed in the great majority of immunocompetent persons, infection with *Mycobacterium tuberculosis* (MTB) is initially contained by the host immune system, resulting in a latent TB infection (LTBI) which is characterized by the presence of immune responses to MTB in the absence of clinical, radiological and microbiological evidences [2]; 5–15% of the infected people develop TB disease during their lifetime, being a reservoir of new active TB cases [1, 3, 4].

The tuberculin skin test (TST) and MTB specific interferon-gamma (IFN-γ) release assays (IGRAs), such as QuantiFERON-TB Gold In-Tube (QFT-G-IT) assay (Cellestis/Qiagen, Carnegie, Australia) and T-SPOT TB assay (Oxford Immunotec, Abingdon, UK) are still the main tools used for the diagnosis of TB infection. Although the newer IGRAs show some improvements over TST [5, 6, 7], neither diagnostic test can differentiate between LTBI, active TB or past TB [8].

Since the reactivation of TB can be averted by preventive treatment, it is important to identify new biomarkers for the diagnosis of LTBI by setting a new *in vitro* diagnostic method for differential diagnosis between active TB and LTBI.

Our group has studied this Ala-DH for more than 25 years and have published, for the first time, its biochemical [9,10], molecular and 3-D structural characterization suggesting modified conformation in latent and active TB [11]. This enzyme has been implicated in adaptation of *M*. *tuberculosis* to anaerobic dormant stage in LTBI. Ala-DH of *M*. *tuberculosis* is a unique enzyme involved in peptidoglycan biosynthesis since it only accepts L-alanine as substrate in contrast to Ala-DH from all other organisms studied, which also use serine as a substrate [9,10]. This enzyme is missing in *M*. *bovis* and in *M*. *bovis* BCG making it highly specific to *M*. *tuberculosis* as the cause of world-wide pulmonary and extra-pulmonary tuberculosis. Even though this protein is present in other pathogenic mycobacteria such as *M*. *marinum* and *M*. *ulcerans*, there is no overlap between symptoms of TB and the low prevalence diseases caused by *M*. *marinum* or *M*. *ulcerans*. The same shall hold good for all other type of microorganisms possessing Ala-DH enzyme. It is worth mentioning that ESAT6 and CFP10 have been used world-wide in the Quantiferon and T-Spot kits and are regarded as specific to *M*. *tuberculosis*, but these antigens are also present in *M*. *marinum* and *M*. *ulcerans*. To the best of our knowledge, there are no reports of significant, false diagnosis by the use of the above mentioned IGRA tests showing that infection and diseases caused by non-tuberculous mycobacteria (NTM) do not pose serious problems as far as cytokine based TB diagnosis is concerned. An extensive, prospective cohort study of 210 BCG- vaccinated neonates in Southern India was published by Dhanasekaran et al. [12] on effects of NTM infection on host biomarkers potentially relevant to TB management. The results showed an up-regulation of IL-2 in children with TB disease but not in NTM subjects confirming our previous report on Ala-DH based IL2 Elispot assay for differential TB diagnosis in children [13].

Given that Ala-DH is not specific for *M*. *tuberculosis* bacilli [14, 15] we cannot exclude that other recombinant proteins would be useful for the setup of other TB diagnostic assays [16].

Although other authors also use IL-2 to detect response in MTB infected subjects [17, 18, 19] we previously showed, for the first time, that out of a number of M. tuberculosis antigens tested including Ala-DH, ESAT6, CFP10, PstS1, HSPX, antigen 85B, only Ala-DH induced IL2 production measured by an ELISPOT assay could clearly distinguish children with LTBI from those with active TB [13]. No other antigen showed this excellent property as far as differential TB diagnosis is concerned [13].

### Aim of the study

This study is focused on improving TB diagnosis by developing a new blood test to discriminate between active and latent TB infection according to the best cost/effectiveness ratio. A new ELISpot test, called LIOSpot® TB, with high specificity and sensitivity was developed based on our previous home-made ELISpot. [13] The new LIOSpot® TB assay, for the first time, provided evidence that MTB Ala-DH antigen was able to stimulate IL-2 production in active TB but not in LTBI.

## Materials and methods

### Patients and definition of study groups

This study was performed on peripheral blood samples collected from patients with confirmed active TB, from subjects diagnosed with LTBI and from healthy donors, who were consecutively enrolled during the period between September 2014 and August 2016 by the Careggi University Hospital, Florence, Italy. Subjects below 18, pregnant women, HIV/AIDS and all those people with any known immunocompromising condition (such as diabetes, hematological malignancies, end stage kidney disease and immunosuppressive therapy) were excluded from the study. None of the healthy patients had recent exposure to active pulmonary TB cases.

Following the approval by the “Area Vasta Centro, Regione Toscana, Ethical Committee” (BIO 14.013), each patient, previously informed of the aim of the study, signed an informed consent.

All subjects were tested with TST (Sanofi Pasteur MSD SNC, Lyon, France) according to the Mantoux method [20] and with the IGRA test QFT-G-IT according to the manufacturer’s instructions [21].

The subjects enrolled in the study were classified as active TB patients, LTBI patients or healthy people in accordance with the current guidelines [2, 22, 23].

Active TB patients, in addition to TST positivity and a generally positive QFT-G-IT that can be negative in a certain number of cases, were detected through clinical, microbiological and radiological findings. The diagnosis' confirmation required MTB identification through fluorescence microscopy, polymerase chain reaction (PCR) or cultural assays upon biological samples, such as sputum or bronchoalveolar lavage (BAL) for respiratory diseases, tissue biopsies, drainage liquid or needle aspirates for extrapulmonary localizations. In particular, all samples were tested with auramin-rhodamine fluorescence microscopic examination to detect acid-alcohol resistant bacilli (BAAR); PCR-based methods like Artus MTB PCR Kit (Qiagen, Venlo, Netherlands) and GeneXpert MTB/RIF assay (Cepheid, Sunnyvale, CA) were used to identify the *Mycobacterium* strain and to detect rifampicine resistance; then mycobacterial-specific solid and liquid culture media were used for the isolation of the infectious agent.

LTBI was diagnosed by a positive test for MTB infection in persons without history of BCG vaccination or by both TST and IGRA positivity in BCG vaccinated subjects, provided the exclusion of active TB by medical history, clinical, radiologic, and microbiologic evaluations [21, 24, 25].

A subject without BCG vaccination and discordant results between TST and QFT-G-IT was attributed to his study group based on the TST result according to the Mantoux method that is still the standard test for LTBI diagnosis; however IGRAs are recommended to confirm LTBI diagnosis in TST positive BCG vaccinated individuals [2, 5, 26]. Patients with assessed TST and/or QFT-G-IT positivity and chest X-ray positivity and/or in the presence of cough (N = 97) were tested for MTB identification through fluorescence microscopy, PCR and cultural assays on biological samples. The MTB identification tests were not performed in subjects with negative TST and negative QFT-G-IT.

Consequently to the diagnosis, the 215 patients were classified as active TB cases (n = 73), LTBI cases (n = 88) and healthy individuals (n = 54). Table 1 summarizes data from the 215 patients enrolled in the study.

**Table 1:** Characteristics of the 215 subjects divided according to the diagnosis in the three study groups.

	Healthy N (%)	LTBI N (%)	Active TB N (%)
	54 (25)	88 (41)	73 (34)
**Age** Median (IQR)	46 (32–63)	46 (35.5–65)	39 (31–61.5)
**Immigrants**	27 (50)	45 (51)	48 (66)
**BCG vaccinated**	21 (38.8)	38 (43.2)	32 (43.8)
**TST (mm)**			
<5	54 (100)	0 (0)	0 (0)
≥5 and <10	0 (0)	5 (5.8)	0(0)
≥10 and <15	0 (0)	13 (14.7)	8 (11)
≥15	0 (0)	70 (79.5)	65 (89)
**QFT-G-IT**			
Negative	50 (92.65)	5 (5.7)	4 (5.48)
Positive	3 (5.5)	80 (90.9)	68 (93.15)
Indeterminate	1 (1.85)	3 (3.4)	1 (1.37)
**Fluorescence microscopy**			
Positive	Not performed	0 (0)	42 (57.5)
Negative	Not performed	24 (100)	31 (42.5)
**PCR-based tests**			
Positive	Not performed	0 (0)	73(100)
Negative	Not performed	24 (100)	0 (0)
**MTB isolation from culture**			
Positive	Not performed	0 (0)	73(100)
Negative	Not performed	24 (100)	0 (0)

### Reagents

Lymphoprep was purchased from Fresenius Kabi Morge AS (Axis-Shield, Oslo, Norway), RPMI 1640 medium from Biochrome (Leonorenstr, Berlin), L-glutamine from Euroclone (Italy), beta-mercaptoethanol from Sigma-Aldrich (Darmstadt, Germany) Na-pyruvate and Non-essential aminoacids from Thermofisher scientific (Waltham, MA, USA), penicillin/streptomycin from PAN biotech (Aidenbach, Germany), fetal bovine serum (HyClone, GE Helathcare Life Science, Utah, USA).

LIOSpot® TB (LIONEX GmbH, Braunschweig, Germany) is an anti-human IL-2 Elispot kit containing *M*. *tuberculosis* ESAT-6, CFP-10 and Ala-DH recombinant antigens, all produced at LIONEX to a purity of more than 98% and endotoxin free; Phytohaemagglutinin mitogen (PHA) was used as a positive control.

### PBMCs isolation

Peripheral blood mononuclear cells (PBMCs) were isolated from blood samples of each enrolled patient, within 8 hours after venipuncture to ensure cell activity, by Ficoll-Hypaque density gradient centrifugation. Briefly, blood was layered on top of Lymphoprep, density gradient of 1.077 g/mL, and centrifuged at 800 x g for 25 minutes at room temperature (15–25°C) without brake. PBMCs layer was harvested and washed two times in PBS, pH 7.4. Cells were counted using the Burker chamber and then transferred into RPMI 1640 complete medium (supplemented with L-glutamine 1%, beta-mercaptoethanol 1%, Na-pyruvate 1%, non-essential aminoacids 1%, penicillin 50.000U and streptomycin 50 mg), with 5% fetal bovine serum to obtain a concentration of 2.5x10^6^ PBMCs/ml.

### Anti-human IL-2 ELISpot assay

The LIOSpot® TB anti-human IL-2 ELISpot kit contains a 96-well plate coated with anti-human IL-2. First we added the positive control (PHA) and the negative control (medium) as single determinations and the three different antigens ESAT-6, CFP-10 and Ala-DH in double determination (5μg/ml); then PBMCs from each patient were seeded in order to have 2.5x10^5^ cells per 0.1ml/well, working under sterile conditions. The plate was incubated in a humidified incubator at 37°C, 5% CO_2_ for 16–24 hours: during incubation time the antigen will activate specific cells to release IL-2 that will be captured by the antibody at the bottom of the well. After the incubation time wells content was discarded and the plate was washed five times with a wash buffer (PBS—0.05% Tween-20) before adding the anti-human IL-2 biotinylated detection antibody to each well. After one hour of incubation, wash steps were repeated and the conjugated horseradish peroxidase (HRP)-streptavidin solution was then added. The plate was incubated again for one hour at room temperature (RT) and, after being washed as already described, TMB (3,3′,5,5′-Tetramethylbenzidine) substrate solution was dispensed into each well and incubated for ten minutes at RT in the dark, until spots were visible. Following the development of spots, the wells' content was discarded and a stop solution was used. The plate was dried and the number of SFC (spot forming cells) was counted by an automated ELISpot reader using the AID EliSpot Software Version 3.2.3.

The SFC count in PHA positive control wells should be more then 50 or show saturation to confirm cellular functionality and vitality. In the negative control it would be expected to have few or no spots; the number of SFC in the negative control is subtracted from the SFC mean value of the wells containing the ESAT-6, CFP-10 and Ala-DH antigens. Results were expressed as number of SFC per million of PBMCs.

### Statistical analysis

Descriptive statistics were used for the calculation of absolute frequencies and percentages for qualitative data, as well as for mean, median (IQR), and standard deviation of quantitative data.

All distributions of ELISpot test results for healthy patients, LTBI subjects or active TB patients were compared using the Mann-Whitney inferential test or the Student t-test.

P <0.05 was considered statistically significant.

Test performance in terms of sensitivity (ability of the test to identify the true positive subjects) and specificity (ability of the test to identify the true negative subjects) was evaluated for each antigen by a ROC (Receiving Operating Characteristic) curve, the elective validation method of a quantitative diagnostic test in a population. The proportion of patients correctly diagnosed, that is the test accuracy, is proportional to the area under the curve (AUC), which can assume values between 0.5 (50% accuracy) and 1 (100% accuracy). According to the classification proposed by Swets the test is not accurate for AUC = 0.5, the test is poorly accurate for 0.5 <AUC≤ 0.7, the test is moderately accurate for 0.7 <AUC≤ 0.9, the test is highly accurate for 0.9 <AUC< 1 and the test is perfect for AUC = 1 [27]. The ROC curve also allows to identify the best cut off value that maximizes the difference between true positive subjects and false positives ones; it is the best threshold value for anti-human IL-2 ELISpot result relative to each specific antigen (ESAT-6, CFP-10 and Ala-DH) in order to discriminate between patients with active TB and subjects with LTBI. To maximize both sensitivity and specificity, the Youden's index (= Sensitivity–[1- Specificity]) can be applied.

Finally, the correlation between the ELISpot diagnostic test for each antigen and the diagnosis performed on each patient using the current methods was calculated by Cohen's Kappa coefficient according to which there would be slight concordance for the value of k = 0.2, poor concordance for the value of K in the range 0.2–0.4, moderate concordance for k value in the range 0.4–0.6, substantial concordance for k value in the range 0.6–0.8, good concordance for k value in the range 0.8–1.

Statistical analysis were performed with SPSS for Windows software version 20.0.

## Results

Two hundred and fifteen subjects were enrolled in this study: 73 patients with active TB, 88 with LTBI and 54 as uninfected controls. We performed LIOSpot® TB according to kit manual on PBMCs isolated from a blood sample of every participant by stimulation with MTB antigens such as ESAT-6, CFP-10 and Ala-DH; the T cell specific response to each antigen was evaluated in terms of IL-2 production. (Table 2).

**Table 2:** IL-2 based ELISpot test results for the 215 enrolled patient.

	Healthy (n = 54)	LTBI (n = 88)	Active TB (n = 73)	P (Healthy vs infected)	P (Latent vs active disease)
**Ala-DH**	0 (0;0)	7.5 (2.5;10)	280 (85;1120)	p≤0.0001	p≤0.0001
**ESAT-6**	2.5 (0;17.5)	180 (30;340)	392,5 (140;1300)	p≤0.0001	p≤0.0001
**CFP-10**	0 (0;15)	192.5 (77.5;660)	480 (325;1590)	p≤0.0001	p≤0.0001

IL-2 based ELISpot test results for the 215 enrolled patients, clustered according to the diagnosis. The values are expressed as mean and interquartile range of SFC per million PBMCs.

Mann-Whitney inferential test or Student t-test were used to compare ELISpot results for IL-2, as SFCs per million PBMCs, between healthy patients, LTBI subjects and active TB ones referring to each tested antigen. Comparing the results of infected and non-infected there were significant differences for all the antigens (p≤0.0001); similarly, comparing the results of patients in LTBI and the group of active TB patients there were significant differences for all three antigens too (p≤0.0001) (Table 2).

ROC curve analysis was performed for each antigen in order to establish the best cut off of the ELISpot test for IL-2 in discriminating between LTBI and active TB.

Considering the response to Ala-DH antigen, a best cut off value of 12.5 SFC per million PBMCs to have a 96% sensitivity and 100% specificity was established. The area under the ROC curve was 0.971 (IC 95%: 0.939–1), so the test was highly accurate in correctly diagnosing subjects with active disease and those with latent infection.

**Fig 1:**
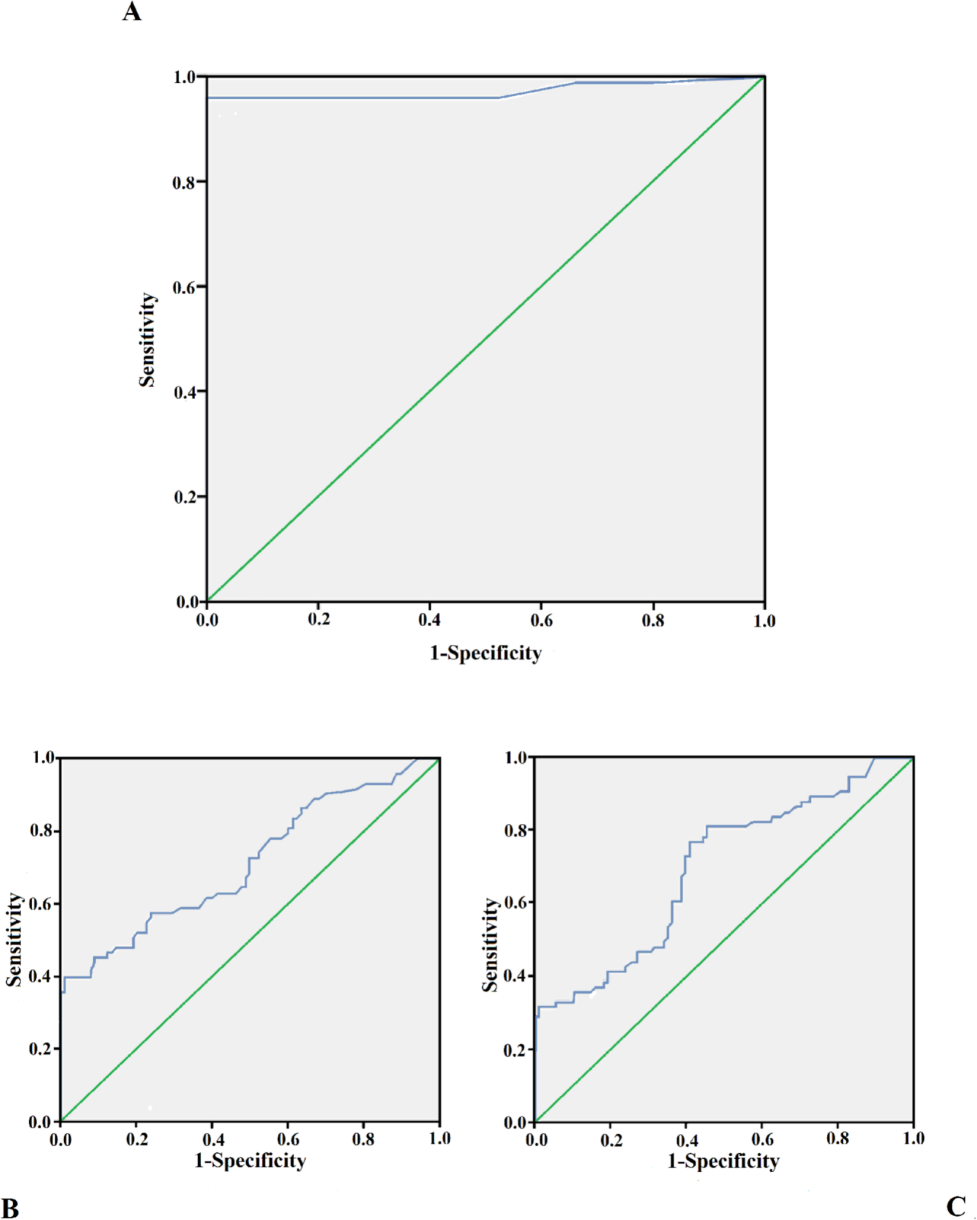
Receiver operator characteristic (ROC) curve. A ROC curve (blu line) plot of the LIOSpot® TB is shown, illustrating sensitivity and specificity for Ala-DH (A), ESAT-6 (B) and CFP-10 (C) to discriminate subjects with active TB from LTBI ones. AUC is the area delimited by the ROC curve and the green reference line.

For the ESAT-6 antigen, with a best cut off of 71.25 SFC per million PBMCs, a sensitivity of 86% and specificity of 36% was obtained. The area under the ROC curve was 0.713 (IC 95%: 0.632–0.794), the test is moderately accurate. Finally, the best cut off for CFP-10 was 231.25 SFC per million PBMCs, with AUC = 0.693 (IC 95%: 0.611–0.774); The test had a low accuracy, with sensitivity of 80% and specificity of 54% (Fig 1).

Once the best threshold value for the ELISpot IL-2 result was set for each specific antigen to discriminate between subjects with active TB and subjects with LTBI, the correlation between ELISpot and TST, that is still the standard test for MTB infection diagnosis, was assessed for every antigen by computing The Cohen's Kappa coefficient and interpreting the data as described above.

The concordance between the ELISpot test for IL-2 and the TST test for all the results obtained in the 215 patients with Ala-DH, ESAT-6 and CFP-10 is: 92.56% (k = 0.82 very good concordance), 77.2% (k = 0.53, moderate concordance) and 63.7% (k = 0.35, poor concordance) respectively (Table 3).

**Table 3:** Concordance between TST and IL-2 based ELISpot for each antigen in the study, for all the enrolled subjects.

TST vs ELISpot Ag	Concordance(%) Cohen’s Kappa (k)
TST vs ELISpot ALA-DH	92.56%, k = 0.82
TST vs ELISpot ESAT-6	77.2%, k = 0.53
TST vs ELISpot CFP-10	63.7%, k = 0.35

The concordances between the different assays are shown in Table 4. The five patients with indetermined Quantiferon were excluded by this statistical analysis. ELISpot test for IL-2 and the QFT-G-IT test for all the results obtained in the 210 patients with Ala-DH is 57.1% (k = 0.25 poor concordance), with ESAT-6 is 79.5% (k = 0.57 moderate concordance) and with CFP-10 is 71% (k = 0.44, moderate concordance). These results are in agreement with results presented in this report since not all QFT-G-IT positive subjects were TB patients.

**Table 4:** Concordance between QFT-G-IT and IL-2 based ELISpot for each antigen in the study, for all the 210 enrolled subjects.

QFT-G-IT vs ELISpot Ag	Concordance(%) Cohen’s Kappa (k)
QFT-G-IT vs ELISpot ALA-DH	57.1%, k = 0.25
QFT-G-IT vs ELISpot ESAT-6	79.5%, k = 0.57
QFT-G-IT vs ELISpot CFP-10	71.0%, k = 0.44

## Discussion

Up to now, there are no biomarkers allowing to differentiate between active TB and LTBI. During LTBI, MTB lives in a non-replicating state, enclosed into the granuloma structure, as long as the host remains immunocompetent; in this period the bacillus still maintains the ability to reactivate and produce active disease when the host immune response is impaired [28, 29].

Studying gene expression profiles and proteomic analysis in both active and quiescent mycobacteria, a number of genes that are differently regulated during the latency phase have been found, in comparison to those expressed during active infection [11, 30]. Different antigens of latency are currently known, several of them were identified in the DosR-regulon [31, 32, 33]. Another gene, Rv2780, that encode L-alanine dehydrogenase (Ala-DH), was found to be over-expressed during the MTB dormancy phase, under nutrient starvation and lack of oxygen regimes [15, 30, 34].

MTB Ala-DH catalyzes reversible conversion of pyruvate to alanine, and glyoxylate to glycine concurrent with the oxidation of NADH to NAD [14] to maintain the optimal NADH/NAD ratio during anaerobiosis in preparation of eventual regrowth, and during the initial response during reoxygenation [15].

Thus MTB Ala-DH was thought to be a useful tool to discriminate between LTBI and active TB [35] and our previous study demonstrates that this antigen is able to stimulate IL-2 production in active TB but not in LTBI in a children population [13]. Given these findings, in the present paper we aimed to confirm our result in an adults cohort tested with the LIOSpot® TB kit, an ELISpot assay developed to detect IL-2 production by T cells stimulated with three MTB antigens, like ESAT-6, CFP-10 (also used in IGRAs) and Ala-DH.

Cytokines play an important role in cell mediated immune responses to MTB infection. The IFN-γ production by activated T cells has been widely considered to play a crucial role in protection against MTB infection [28].

In the last decade, IGRAs became a landmark in the diagnosis of MTB infection. However, these assays do not discriminate between latent infection and active tuberculosis disease, as TST.

The choice of setting the LIOSpot® TB assay on IL-2 cytokine production is due to the fact that recent studies demonstrate the importance of this cytokine to discriminate between latent and active tuberculosis infection, thus being a new possible diagnostic biomarker [35, 36, 37, 38]; based on the fact that IL-2 is significantly differentially produced by individuals with LTBI and active TB patients [37, 39, 40].

According to these findings, LIOSpot® TB was set as an anti human IL-2 ELISpot assay able to detect IL-2 production after PBMCs stimulation with ESAT-6, CFP-10 and Ala-DH of MTB.

In our study, all the healthy subjects showed no significant production of IL-2 (SFC per million of PBMCs) in response to T cell stimulation by the three different antigens: Ala-DH (0: 0–0), ESAT-6 (2.5: 0–17.5) and CFP-10 (0: 0–15). Furthermore it is of note that none of the 21 BCG-vaccinated healthy subjects gave positive IL-2 responses to Ala-DH. The LIOSpot® TB detected a significant production of IL-2 after stimulation with ESAT-6 (392.5: 140–1300), CFP-10 (480: 325–1590) and with Ala-DH (280: 85–1120) in 73 patients group with active TB. Among people in the LTBI group, despite the smaller but substantial production of IL-2 in response to ESAT-6 and CFP-10 stimulation (180: 30–340 and 192.5: 77.5–660 SFC per million of PBMCs respectively) compared to active TB cases, Ala-DH induced a very low amount of IL-2 production by stimulated T cells (7.5: 2.5–10 SFC per million of PBMCs).

Even though comparing the LIOSpot® TB results for each tested antigen between uninfected and infected subjects and between people with LTBI and active TB, all the differences were significant (p< 0.0001), the ROC analysis demonstrated a high accuracy of the test only for Ala-DH: with a cut off value of 12.5 SFC per million PBMCs, the Ala-DH ROC curve conferred 96% sensitivity and 100% specificity to the test. Thus, the LIOSpot® TB test is highly accurate and is able to make a differential diagnosis between subjects with active TB and those with LTBI.

For the ESAT-6 antigen, with a best cut off value of 71.25 SFC per million PBMCs, a sensitivity of 86% and specificity of 36% was obtained. Finally, the best cut off value for CFP-10 was 231.25 SFC per million PBMCs, with sensitivity of 80% and specificity of 54%.

Despite the low specificity, these thresholds confer to the LIOSpot® TB the ability to detect true tuberculosis infection.

Overall, the present study demonstrates that the LIOSpot® TB test is a very useful diagnostic tool to discriminate between LTBI and active tuberculosis infection.

## Supporting information

S1 File ELISpot supporting information. IL-2 based ELISpot results for each of the active TB, LTBI patients and healthy subjects.
(PDF)
