# Supplementary material for: Novel M. tuberculosis specific IL-2 ELISpot assay discriminates adult patients with active or latent tuberculosis
Source: PLoS One. 2018 Jun 1;13(6):e0197825. doi: 10.1371/journal.pone.0197825 (PMC5983515; doi:10.1371/journal.pone.0197825)
Supplement: S1 File — IL-2 based ELISpot results for each of the active TB, LTBI patients and healthy subjects. (PDF) [file pone.0197825.s001.pdf]

ELISpot Supporting information

IL-2 based ELISpot results for each of the active TB, LTBI patients and healthy subjects.

IL-2 based ELISpot test results for the 215 enrolled patient: IL-2 based ELISpot test results for the 215 enrolled patients, clustered according to the diagnosis. The values are expressed as mean and interquartile range of SFC per million PBMCs.

	**Healthy (n=54)**	**LTBI (n=88)**	**Active TB (n=73)**	**P (Healthy vs infected)**	**P (Latent vs active disease)**
**Ala-DH**	0 (0;0)	7.5 (2.5;10)	280 (85;1120)	p≤0.0001	p≤0.0001
**ESAT-6**	2.5 (0;17.5)	180 (30;340)	392,5 (140;1300)	p≤0.0001	p≤0.0001
**CFP-10**	0 (0;15)	192.5 (77.5;660)	480 (325;1590)	p≤0.0001	p≤0.0001

ELISpot images and spot number for HEALTHY SUBJECTS (N=54)

N	Medium (negative control, **subtracted**) spot/ 0.2 x 10^6 cell	Medium Pic spot/ 0.2 x 10^6 cell	Ala-DH pic spot/ 0.2 x 10^6 cell	Ala-DH spot/ 0.2 x 10^6 cell	Ala-DH spot/ 10^6 cell	ESAT-6 pic spot/ 0.2 x 10^6 cell	ESAT-6 spot/ 0.2 x 10^6 cell	ESAT-6 spot/ 10^6 cell	CFP-10 pic spot/ 0.2 x 10^6 cell	CFP-10 spot/ 0.2 x 10^6 cell	CFP-10 spot/ 10^6 cell	PHA (positive control) spot/ 0.2 x 10^6 cell	PHA pic spot/ 0.2 x 10^6 cell
1	0	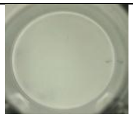	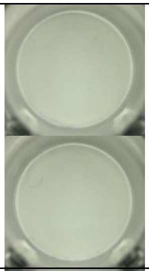	0	0	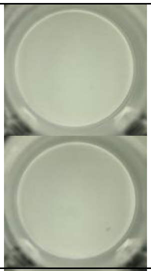	0	0	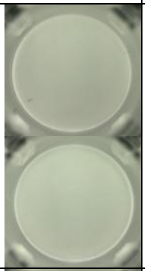	0	0	257	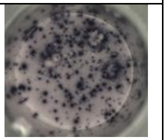
2	0	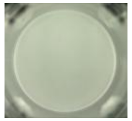	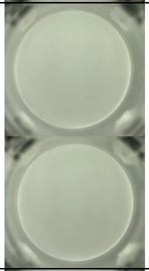	0	0	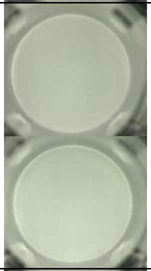	0	0	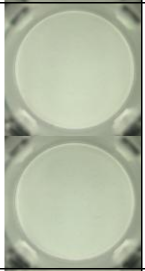	0	0	105	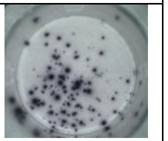
3	0	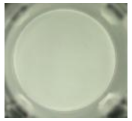	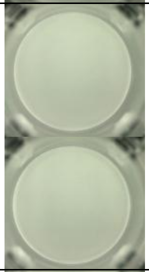	0	0	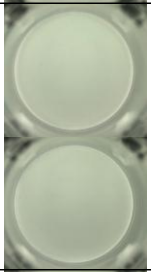	0	0	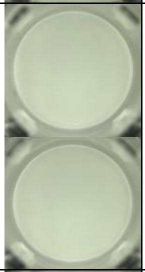	0	0	315	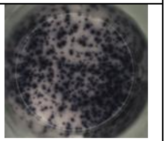
4	0	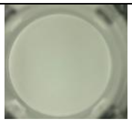	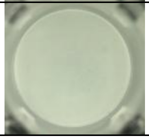	0	0	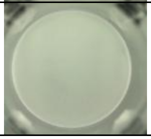	0	0	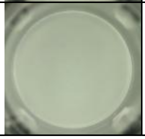	0	0	267	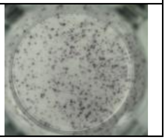

			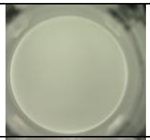			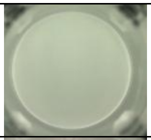			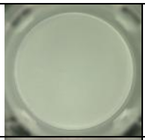				
5	0	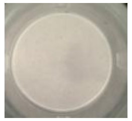	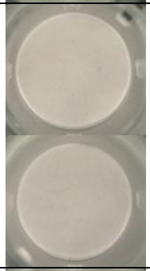	0	0	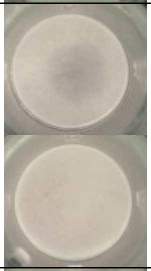	0	0	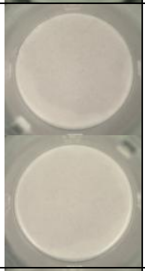	0	0	248	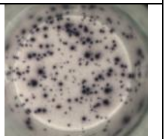
6	0	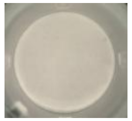	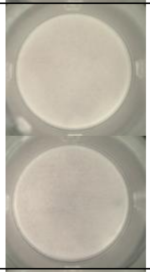	0	0	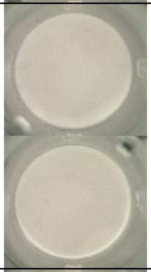	0	0	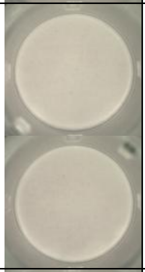	0	0	271	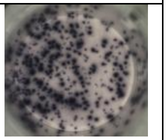
7	0	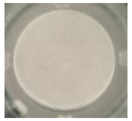	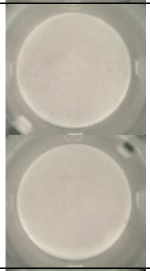	0	0	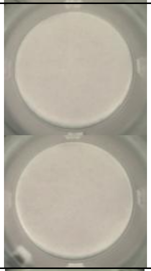	0	0	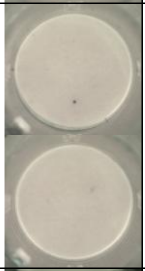	(1+1)<br>1	5	265	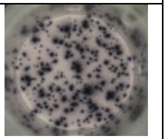
8	0	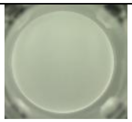	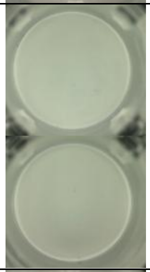	0	0	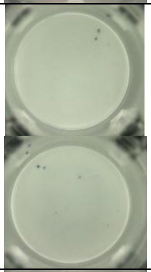	4	20	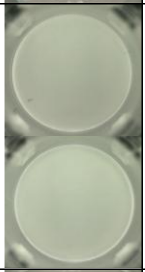	(2+2)<br>2	10	87	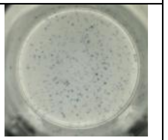
9	0	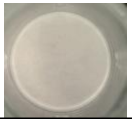	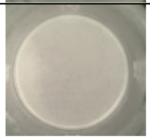	0	0	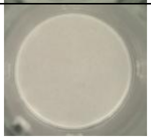	0	0	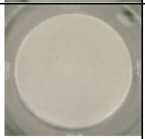	0	0	182	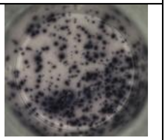

			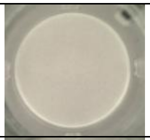			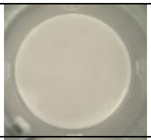			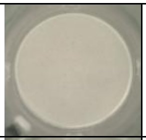				
10	0	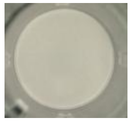	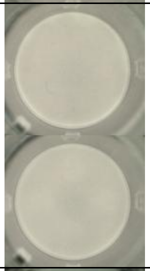	0	0	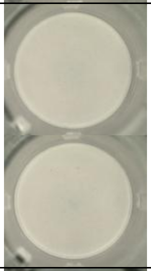	0	0	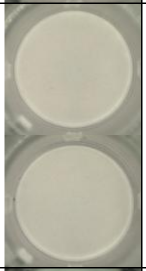	0	0	194	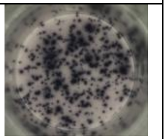
11	0	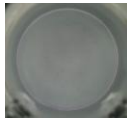	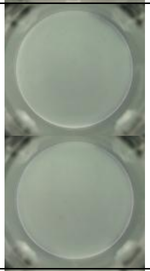	0	0	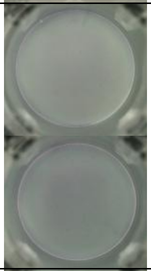	0	0	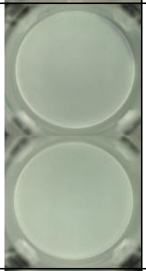	0	0	292	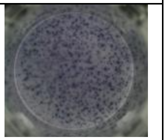
12	0	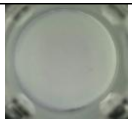	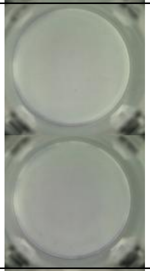	0	0	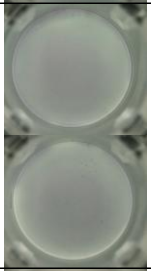	0	0	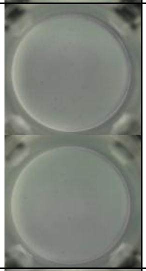	0	0	270	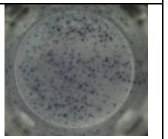
13	0	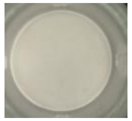	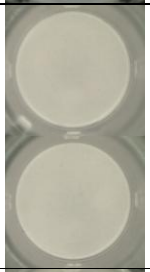	0	0	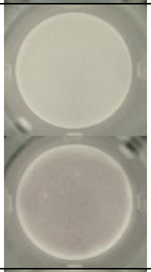	0	0	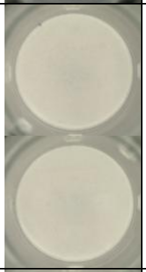	0	0	201	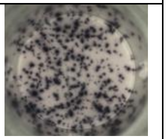
14	0	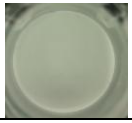	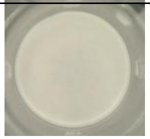	0	0	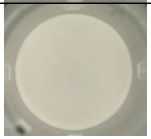	0	0	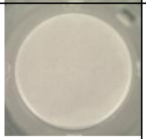	0	0	215	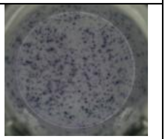

			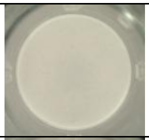			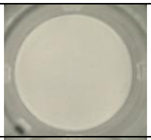			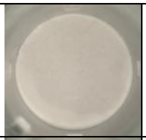				
15	0	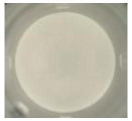	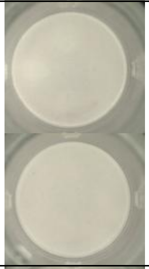	0	0	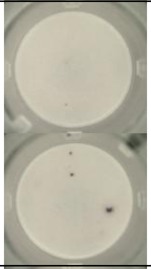	(3+1) 2	10	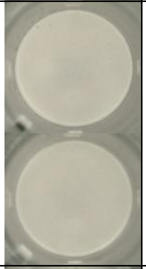	(2+0) 1	5	230	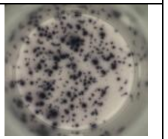
16	0	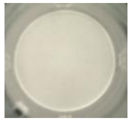	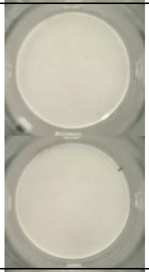	0	0	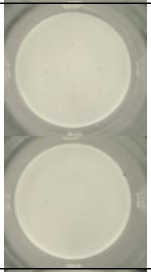	0	0	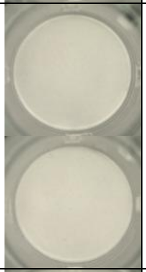	0	0	254	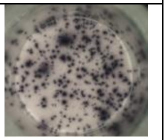
17	0	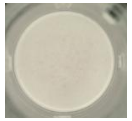	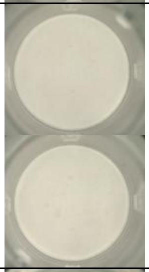	0	0	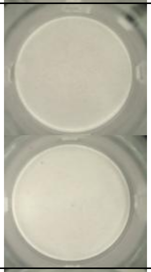	(2+0) 1	5	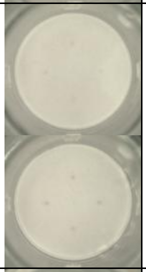	0	0	296	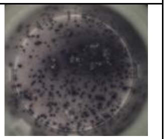
18	0	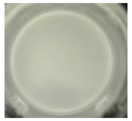	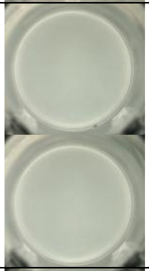	0	0	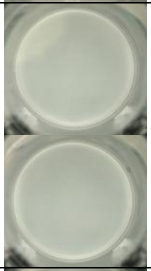	0	0	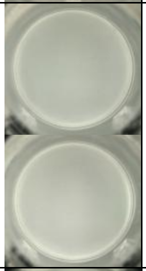	0	0	208	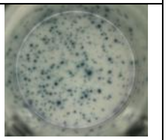
19	0	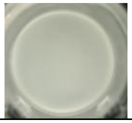	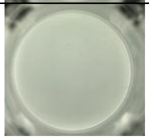	0	0	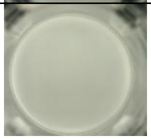	0	0	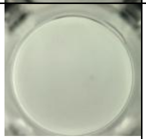	0	0	217	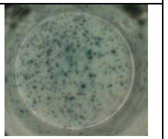

			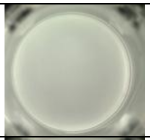			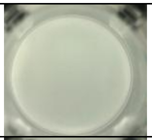			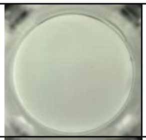				
20	0	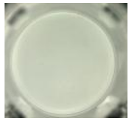	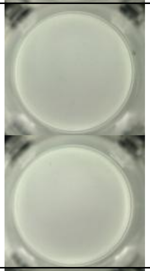	0	0	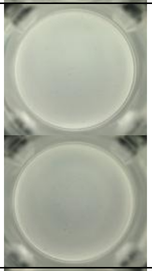	0	0	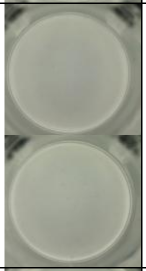	0	0	146	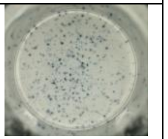
21	0	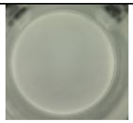	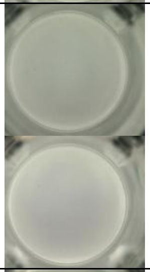	0	0	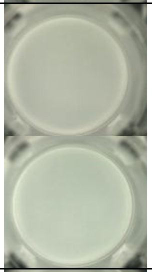	0	0	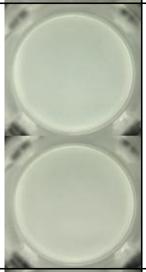	0	0	182	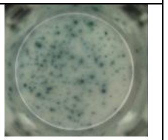
22	0	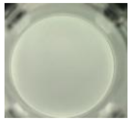	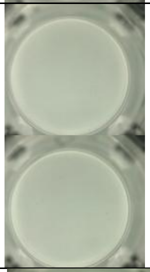	0	0	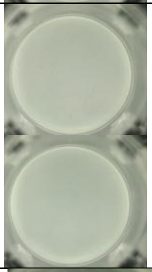	0	0	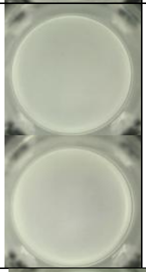	0	0	79	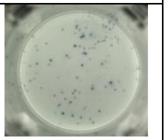
23	0	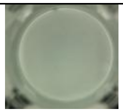	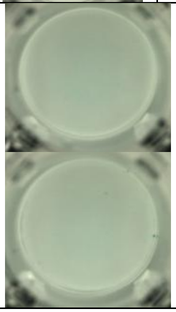	0	0	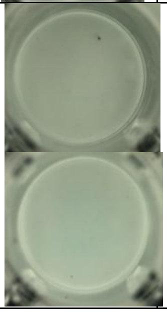	(0+2) 1	5	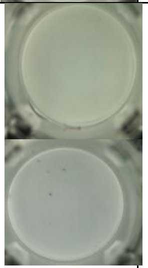	(2+2) 2	10	197	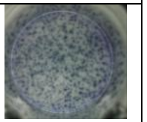

24	0	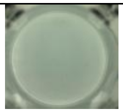	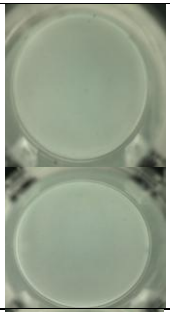	0	0	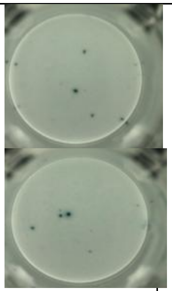	(5+7)<br>6	30	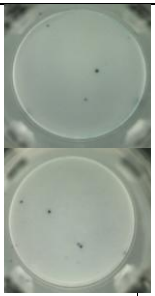	(4+3)<br>3,5	17,5	113	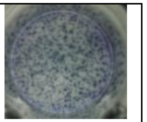
25	0	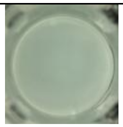	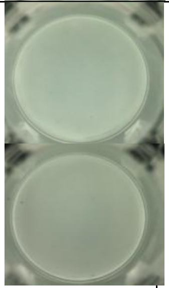	(1+0)<br>0,5	2,5	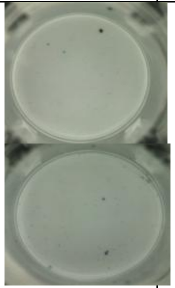	(4+2)<br>3	15	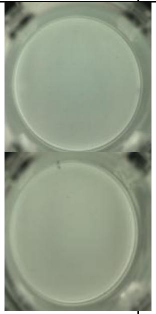	(0+0)<br>0	0	218	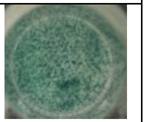
26	0	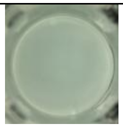	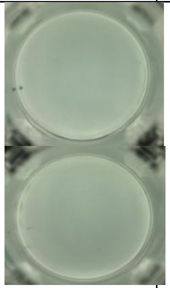	0	0	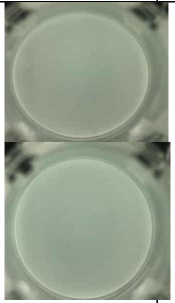	0	0	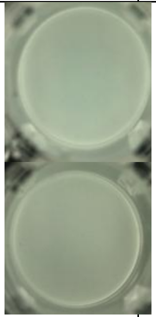	(0+0)<br>0	0	296	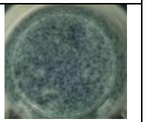
27	0	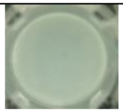	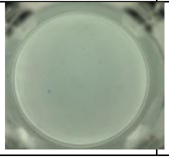	0	0	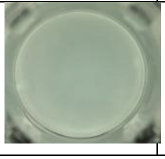	0	0	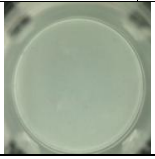	(1+0)<br>0,5	2,5	203	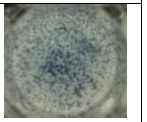

			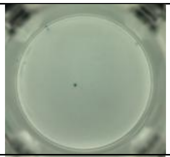			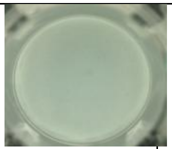			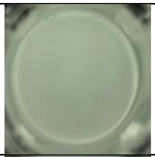				
28	0	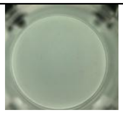	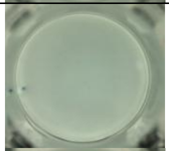 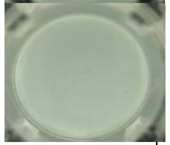	0	0	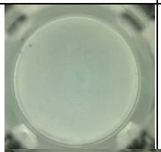 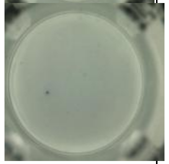	(2+1) 1,5	7,5	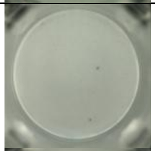 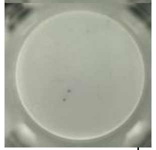	(3+1) 2	10	206	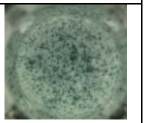
29	0	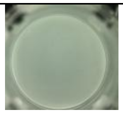	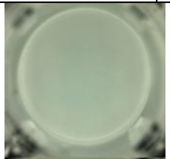 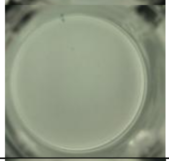	0	0	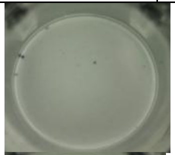 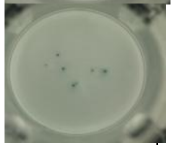	(4+6) 5	25	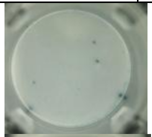 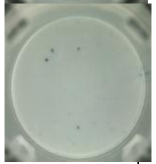	(5+4) 4,5	22,5	243	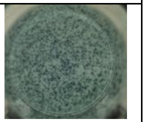
30	0	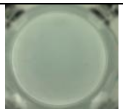	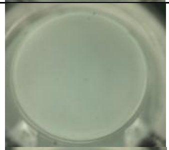 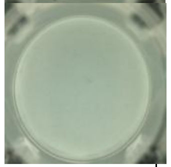	0	0	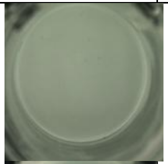 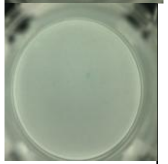	0	0	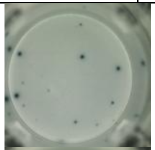 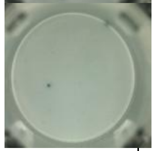	(7+5) 6	30	261	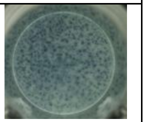

31	0	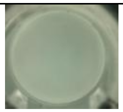	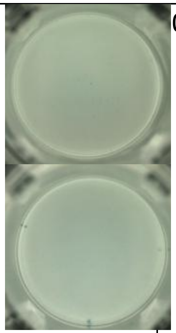	0	0	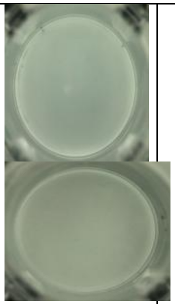	0	0	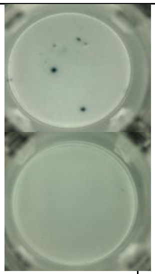	(3+11) 7	35	235	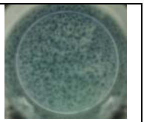
32	0	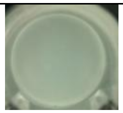	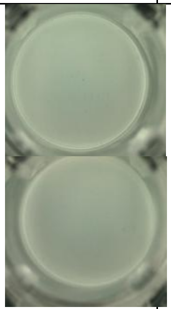	0	0	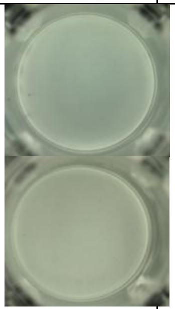	(0+1) 0,5	2,5	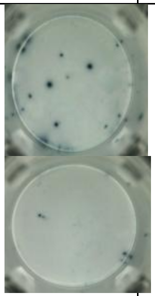	(10+8) 9	45	265	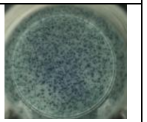
33	0	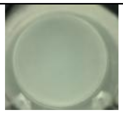	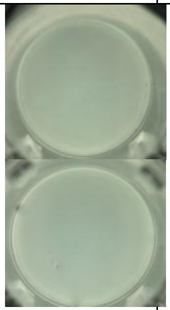	0	0	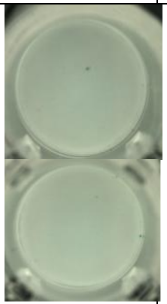	(1+1) 1	5	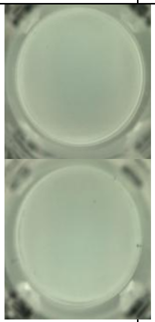	(0+0) 0	0	231	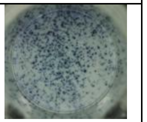
34	0	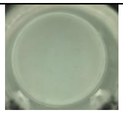	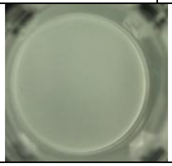	0	0	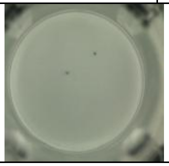	(3+1) 2	10	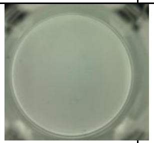	(0+0) 0	0	198	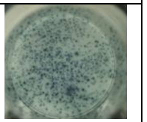

			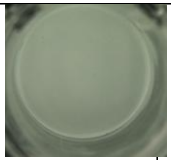			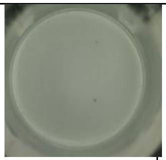			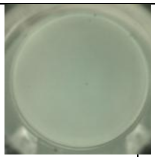				
35	0	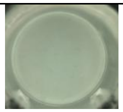	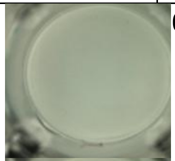<br>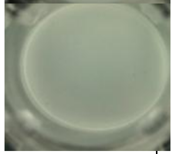	0	0	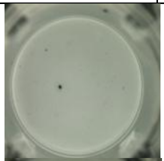<br>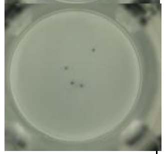	(4+3)<br>3,5	17,5	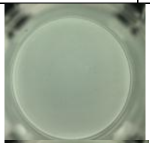<br>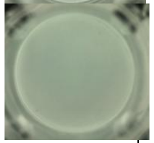	(0+0)<br>0	0	203	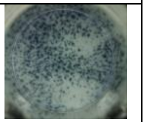
36	0	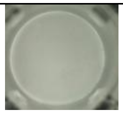	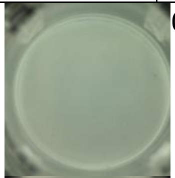<br>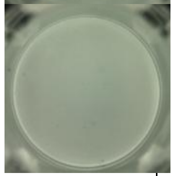	0	0	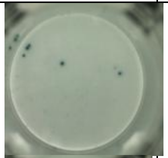<br>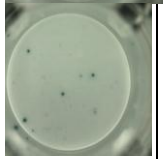	(6+8)<br>7	35	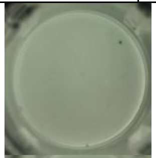<br>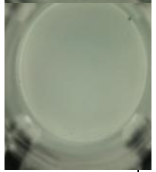	(1+0)<br>0,5	2,5	295	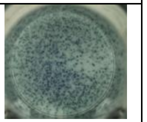
37	0	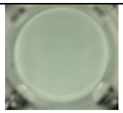	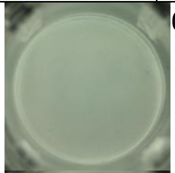<br>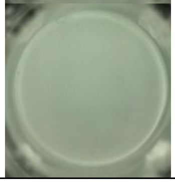	0	0	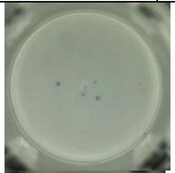<br>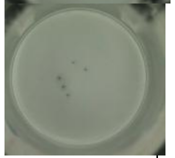	(6+4)<br>5	25	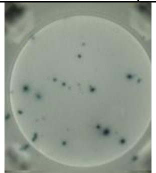<br>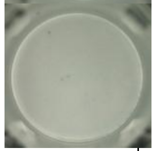	(13+9)<br>11	55	287	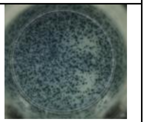

38	0	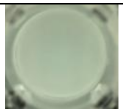	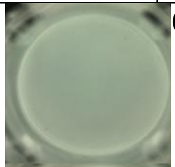 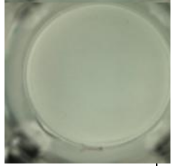	0	0	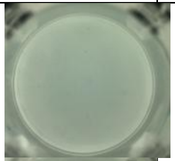 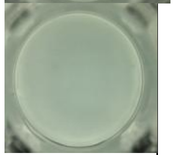	0	0	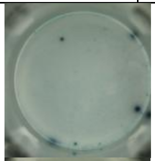 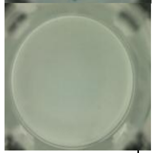	(4+6) 5	25	223	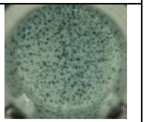
39	0	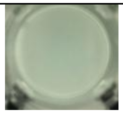	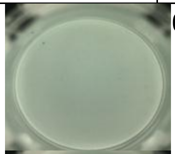 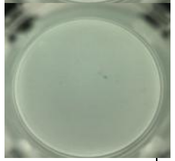	0	0	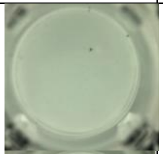 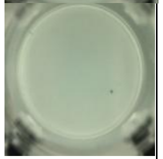	(1+1) 1	5	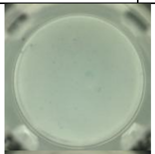 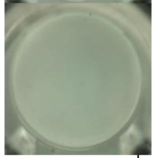	(0+0) 0	0	243	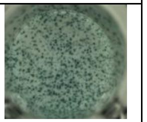
40	0	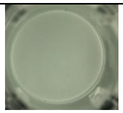	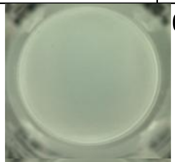 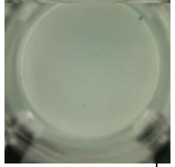	0	0	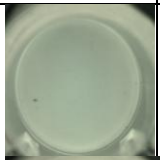 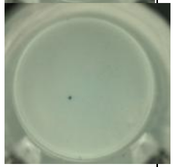	(1+1) 1	5	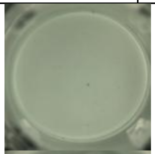 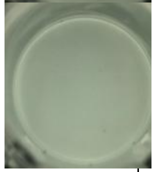	(1+0) 0,5	2,5	252	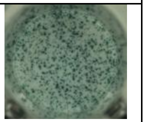

41	0	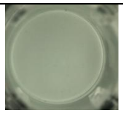	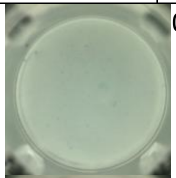 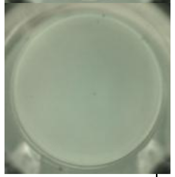	0	0	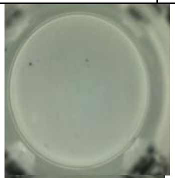 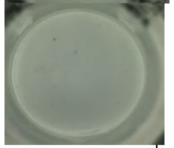	(2+2) 2	10	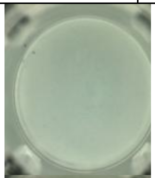 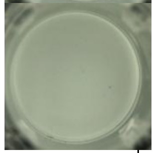	(1+1) 1	5	301	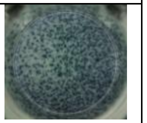
42	0	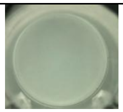	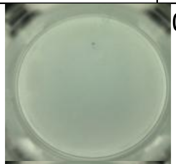 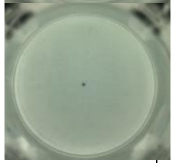	0	0	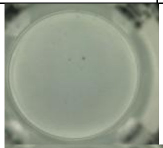 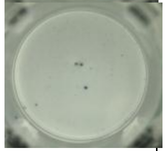	(5+3) 4	20	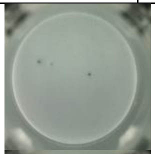 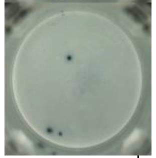	(1+1) 1	5	297	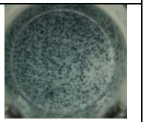
43	0	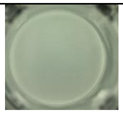	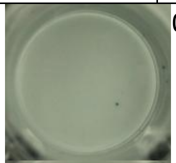 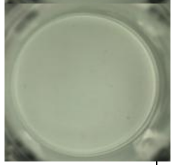	0	0	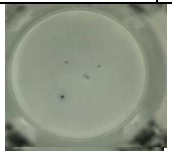 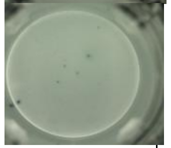	(6+5) 5,5	27,5	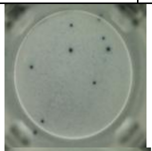 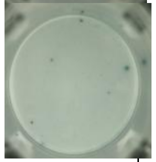	(7+6) 6,5	32,5	282	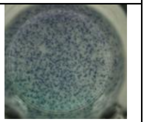

44	0	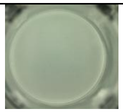	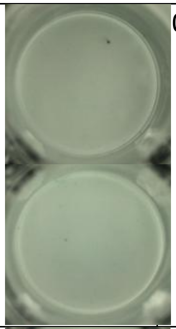	0	0	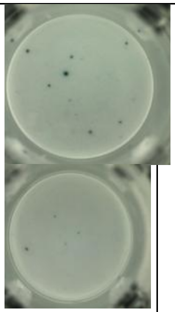	(5+7)<br>6	30	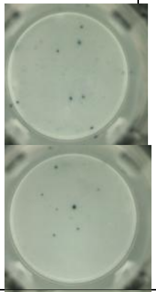	(6+8)<br>7	35	261	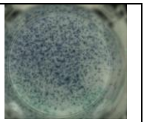
45	0	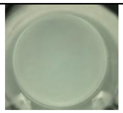	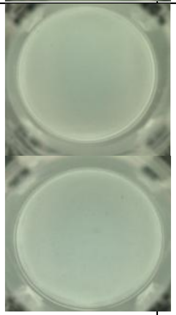	(0+1)<br>0,5	2,5	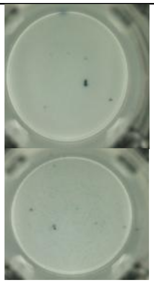	(6+8)<br>7	35	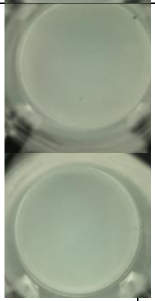	(0+0)<br>0	0	204	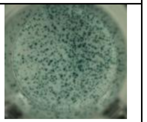
46	0	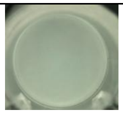	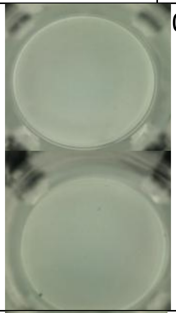	0	0	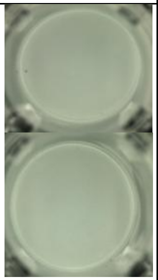	(1+0)<br>0,5	2,5	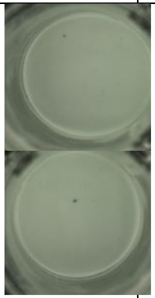	(0+0)<br>0	0	215	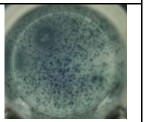
47	0	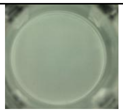	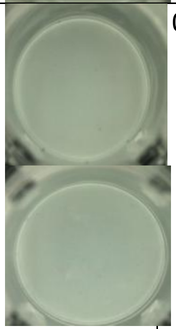	0	0	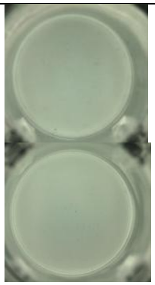	(1+1)<br>1	5	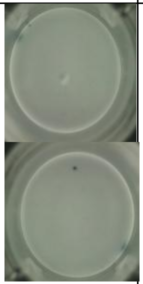	(1+1)<br>1	5	197	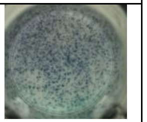

48	0	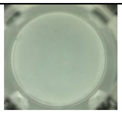	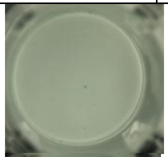 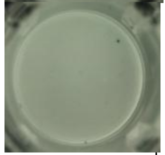	0	0	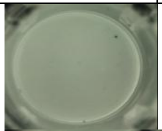 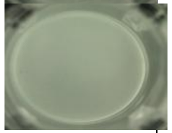	(1+0)<br>0,5	2,5	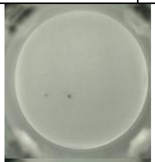 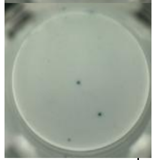	(2+3)<br>2,5	12,5	196	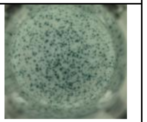
49	0	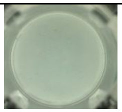	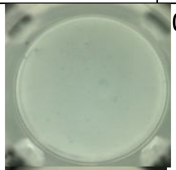 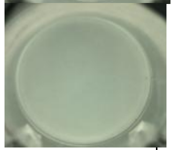	0	0	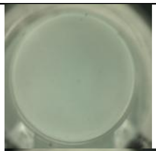 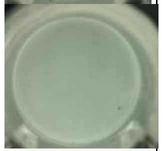	(1+1)<br>1	5	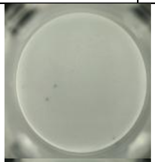 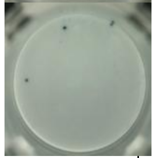	(3+2)<br>2,5	12,5	190	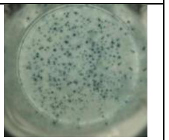
50	0	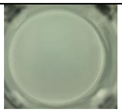	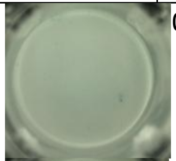 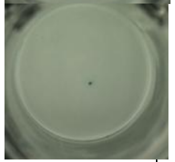	0	0	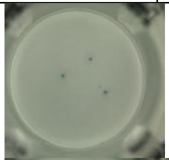 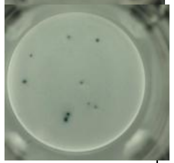	(6+7)<br>6,5	32,5	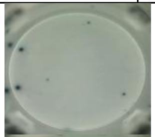 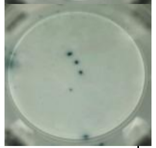	(4+6)<br>5	25	204	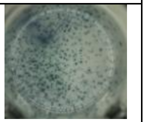

51	0	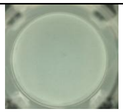	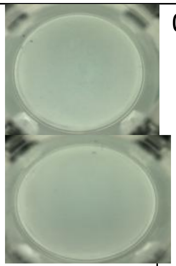	0	0	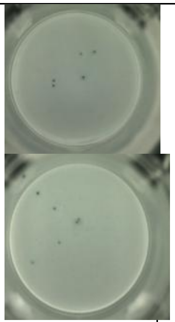	(6+5)<br>5,5	27,5	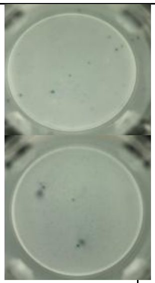	(5+8)<br>6,5	32,5	265	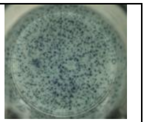
52	0	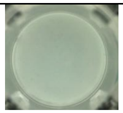	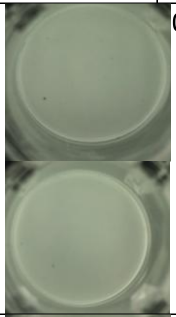	0	0	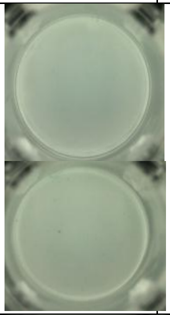	(1+0)<br>0,5	2,5	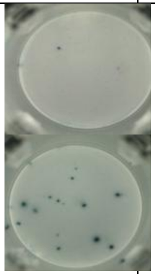	(10+6)<br>8	40	271	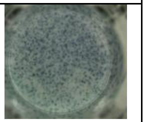
53	0	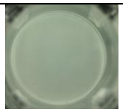	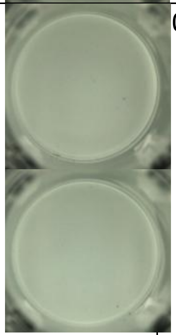	0	0	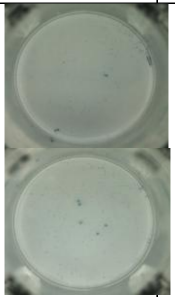	(5+3)<br>4	20	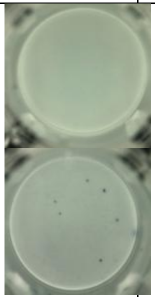	(4+2)<br>3	15	283	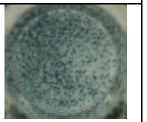
54	0	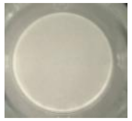	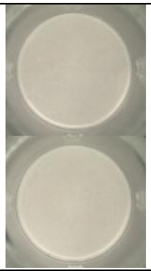	0	0	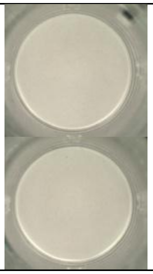	(0+2)<br>1	0	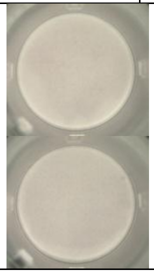	0	0	217	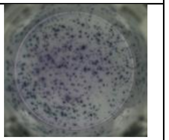

ELISpot images and spot number for LTBI SUBJECTS (N=88)

N	Medium (negative control, subtracted) spot/ 0.2 x 10^6 cell	Medium Pic spot/ 0.2 x 10^6 cell	Ala-DH pic spot/ 0.2 x 10^6 cell	Ala-DH spot/ 0.2 x 10^6 cell	Ala-DH spot/ 10^6 cell	ESAT-6 pic spot/ 0.2 x 10^6 cell	ESAT-6 spot/ 0.2 x 10^6 cell	ESAT-6 spot/ 10^6 cell	CFP-10 pic spot/ 0.2 x 10^6 cell	CFP-10 spot/ 0.2 x 10^6 cell	CFP-10 spot/ 10^6 cell	PHA (positive control) spot/ 0.2 x 10^6 cell	PHA pic spot/ 0.2 x 10^6 cell
1	0	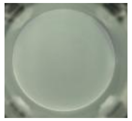	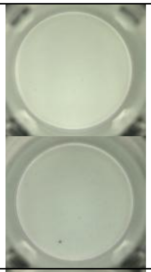	(1+1) 1	5	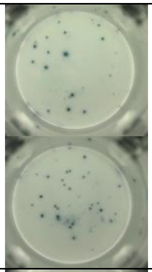	(24+36) 30	150	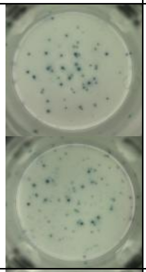	(65+55) 60	300	178	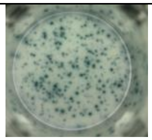
2	0	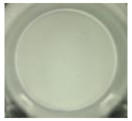	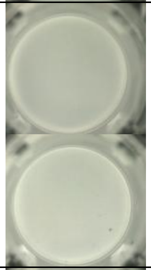	(0+1) 0,5	2,5	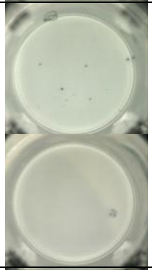	(3+6) 4,5	22,5	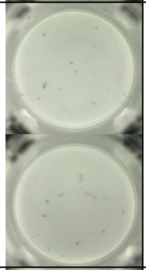	(19+12) 15,5	77,5	198	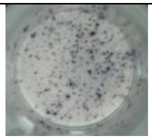
3	2	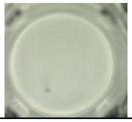	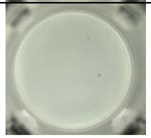	(2+2) 0	0	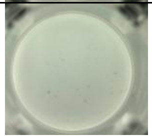	(8+8) 6	30	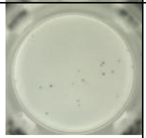	(15+13) 12	60	214	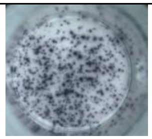

			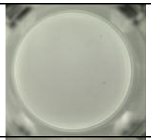			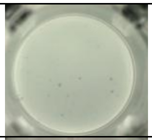			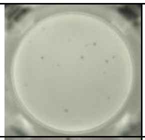				
4	1	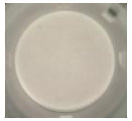	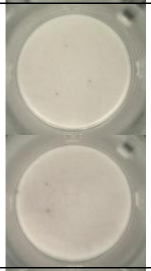	(2+3)<br>1,5	7,5	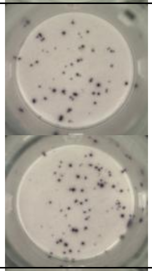	(66+52)<br>58	290	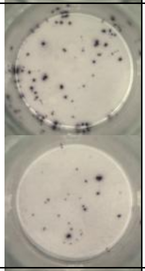	(47+29)<br>37	185	195	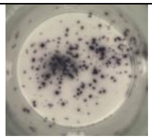
5	0	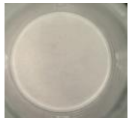	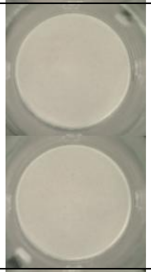	0	0,5	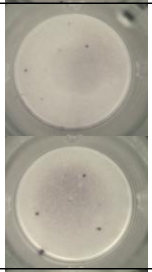	(7+5)<br>6	30	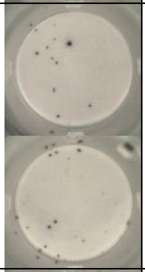	(13+17)<br>15	75	174	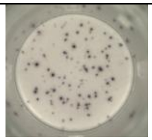
6	1	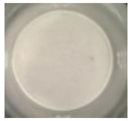	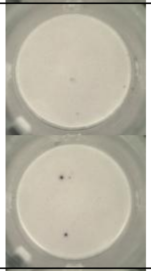	(3+3)<br>2	10	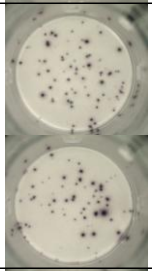	(75+61)<br>67	335	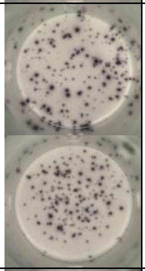	(152+162)<br>156	780	196	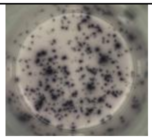
7	0	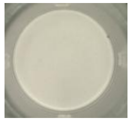	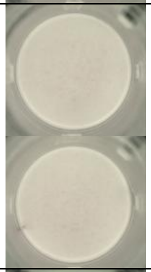	(0+1)<br>0,5	2,5	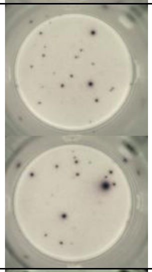	(19+17)<br>18	90	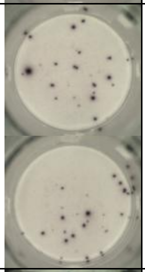	(24+25)<br>24,5	122	187	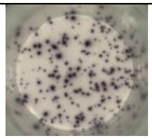
8	4	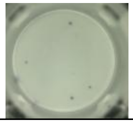	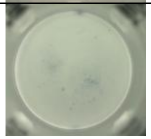	(7+5)<br>2	10	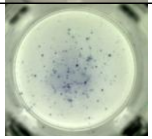	(86+90)<br>84	420	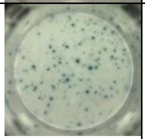	(138+130)<br>130	650	268	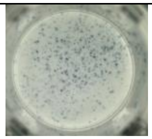

			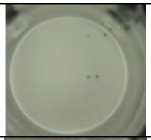			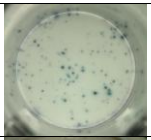			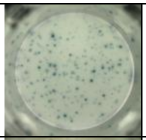				
9	0	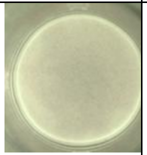	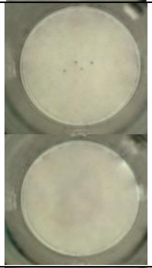	(4+0)<br>2	10	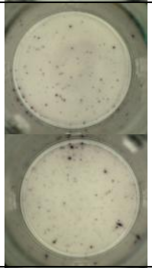	(57+65)<br>61	305	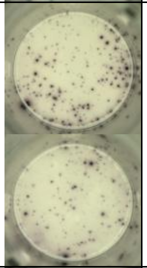	(124+116)<br>112	560	214	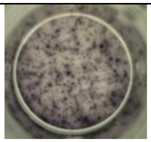
10	0	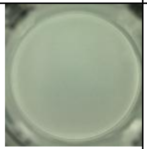	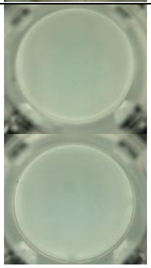	(0+0)<br>0	0	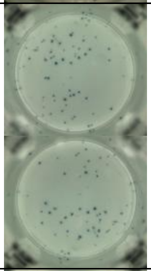	(68+66)<br>67	335	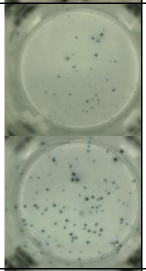	(86+42)<br>64	320	284	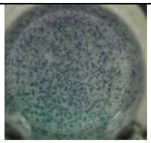
11	0	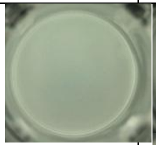	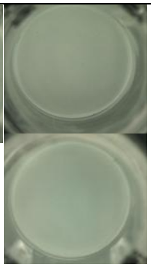	(0+0)<br>0	0	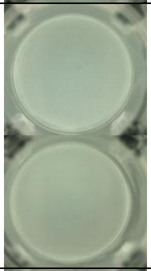	0	0	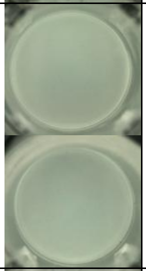	0	0	264	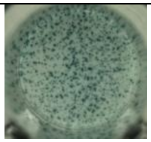
12	2	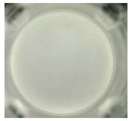	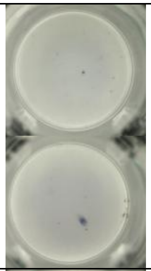	(3+5)<br>2	10	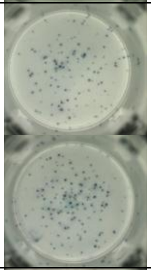	(105+127)<br>114	570	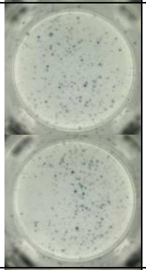	(176+196)<br>184	920	247	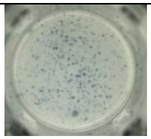
13	0	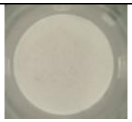	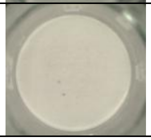	(1+2)<br>1,5	7,5	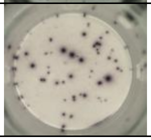	(73+53)<br>63	315	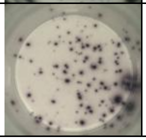	(107+85)<br>96	480	256	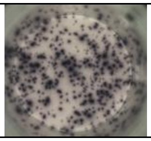

			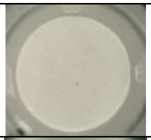			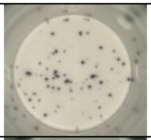			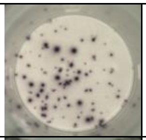				
14	0	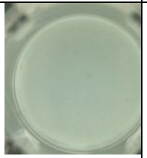	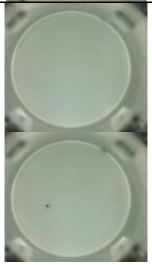	(0+1)<br>0,5	2,5	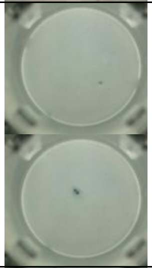	(2+1)<br>1,5	7,5	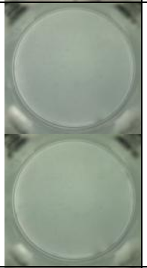	(0+0)<br>0	0	259	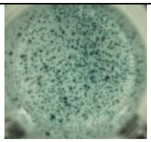
15	2	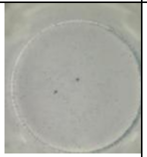	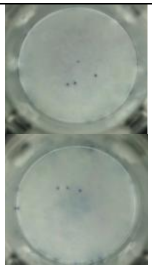	(4+4)<br>2	10	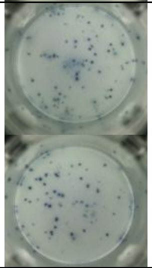	(82+74)<br>78	390	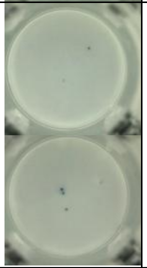	(4+2)<br>1	5	246	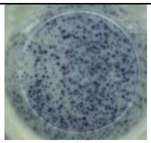
16	0	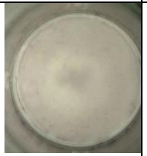	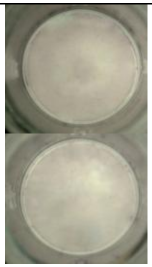	(0+0)<br>0	0	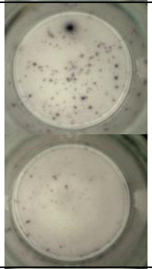	(95+71)<br>83	415	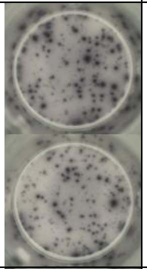	(141+153)<br>147	735	247	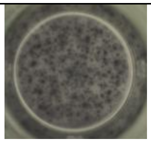
17	0	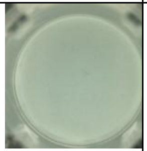	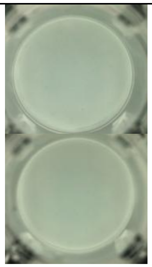	(0+0)<br>0	0	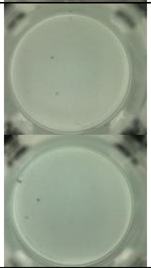	(3+3)<br>3	15	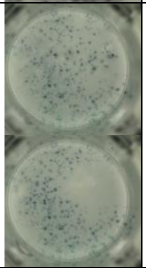	(175+167)<br>171	835	283	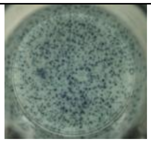
18	0	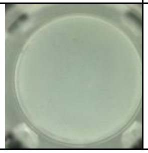	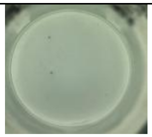	(2+2)<br>2	10	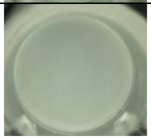	(0+0)<br>0	0	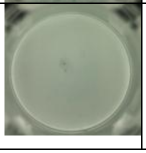	(0+0)<br>0	0	291	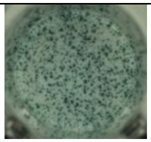

			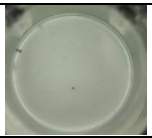			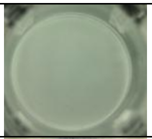			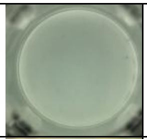				
19	0	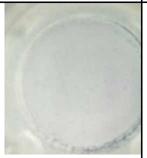	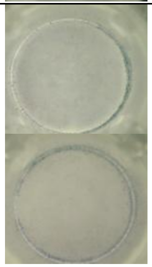	(0+0)<br>0	0	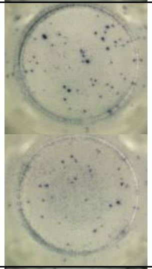	(67+63)<br>65	325	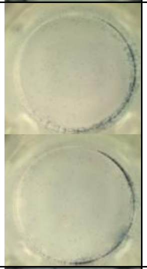	(0+0)<br>0	0	304	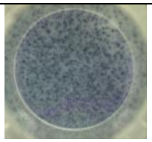
20	0	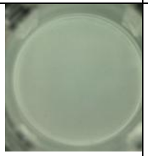	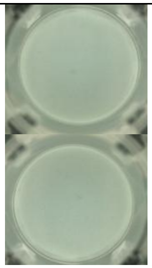	(0+0)<br>0	10	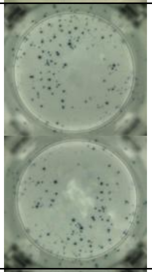	(90+86)<br>88	440	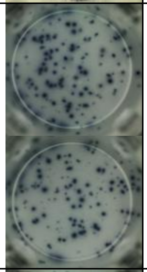	(86+112)<br>99	495	315	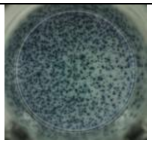
21	0	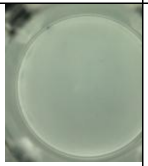	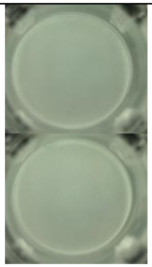	(0+0)<br>0	0	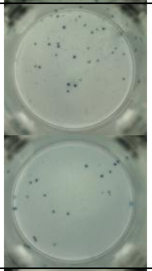	(25+23)<br>24	120	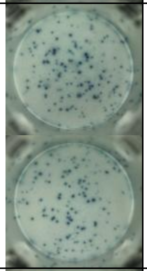	(116+124)<br>120	600	305	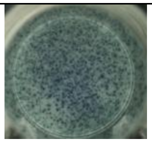
22	0	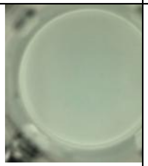	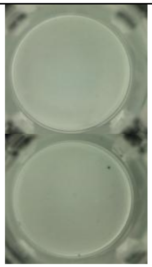	(0+1)<br>0,5	2,5	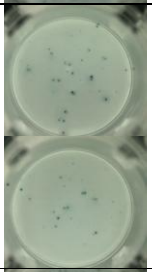	(28+26)<br>27	135	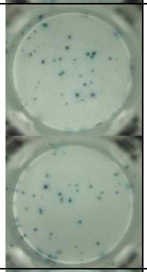	(30+33)<br>31,5	157,5	285270	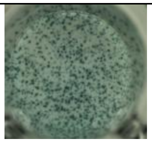
23	4	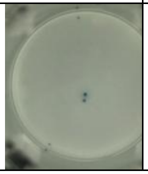	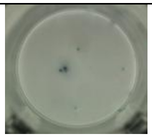	(6+6)<br>2	10	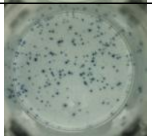	(154+16<br>2)154	770	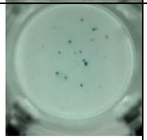	(20+60)<br>36	180		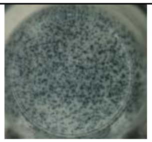

			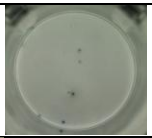			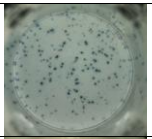			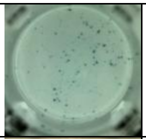				
24	0	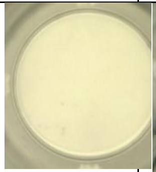	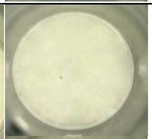<br>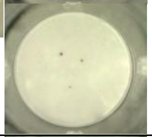	(1+3)<br>2	10	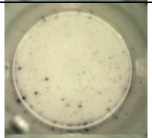<br>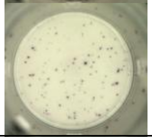	(81+79)<br>80	400	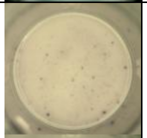<br>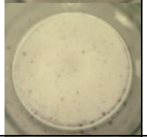	(45+30)<br>37,5	187,5	292	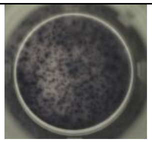
25	0	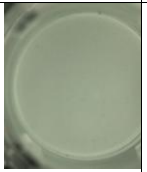	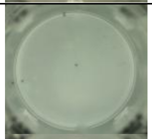<br>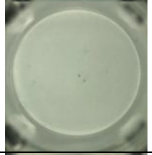	(2+2)<br>2	10	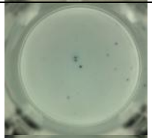<br>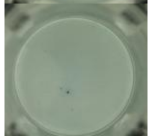	(3+9)<br>6	30	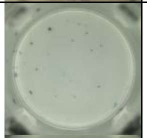<br>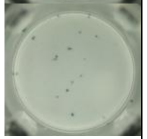	(15+16)<br>15,5	77,5	284	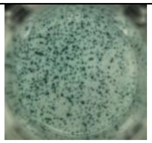
26	0	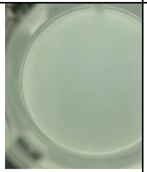	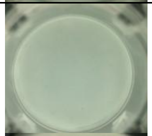<br>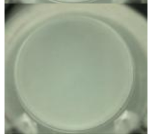	(0+0)<br>0	0	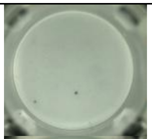<br>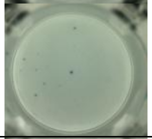	(8+8)<br>8	40	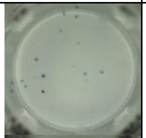<br>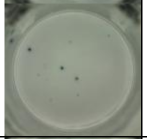	(8+14)<br>11	55	306	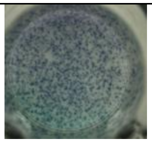
27	0	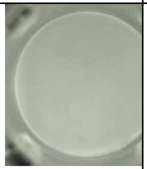	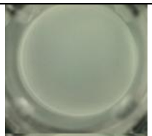<br>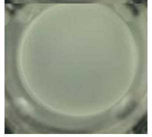	(0+0)<br>0	0	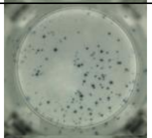<br>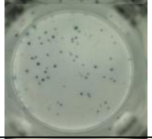	(87+85)<br>86	430	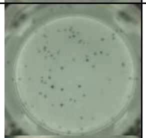<br>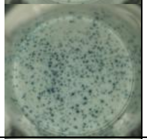	(379+55)<br>217	1085	312	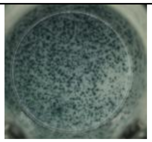
28	0	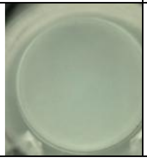	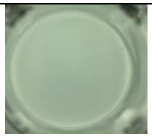	(0+0)<br>0	0	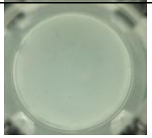	(0+0)<br>0	0	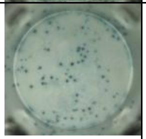	(194+158)<br>176	880	295	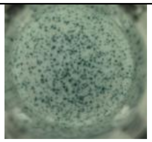

			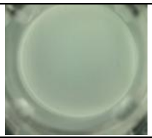			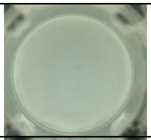			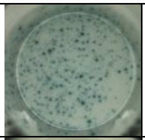				
29	0	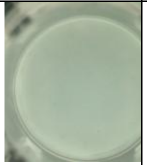	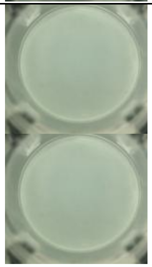	(0+0)<br>0	0	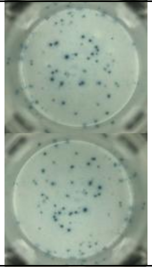	(60+56)<br>58	290	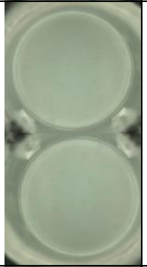	(0+0)<br>0	0	271	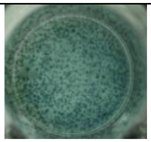
30	0	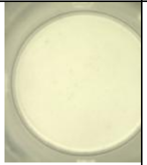	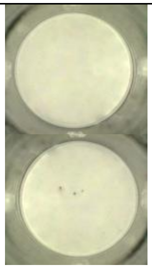	(4+0)<br>2	10	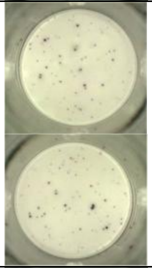	(60+65)<br>62,2	311	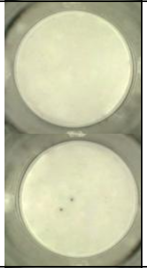	(2+0)<br>1	5	276	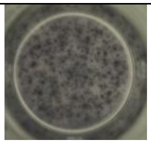
31	0	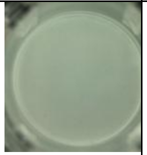	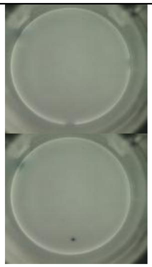	(0+1)<br>0,5	2,5	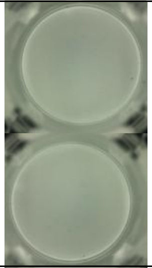	(0+1)<br>0,5	2,5	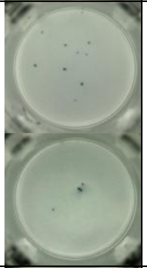	(4+10)<br>7	35	325	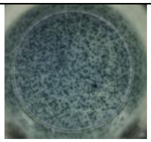
32	0	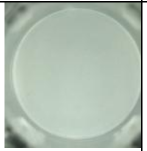	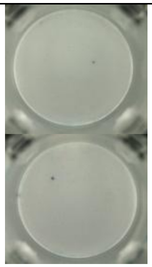	(1+1)<br>1	5	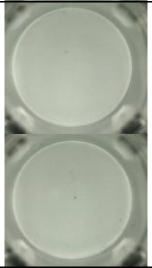	(2+2)<br>2	10	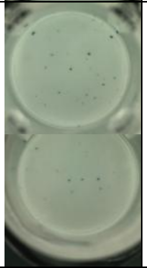	(17+11)<br>14	70	294	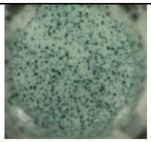
33	0	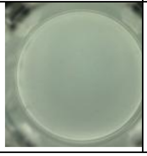	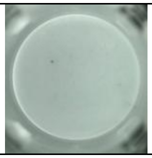	(1+1)<br>1	5	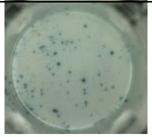	(85+79)<br>82	410	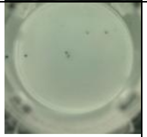	(40+5)<br>22,5	112,5	283	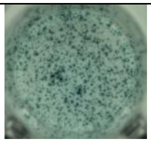

			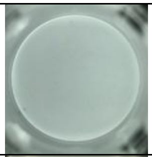			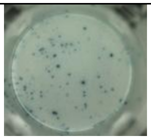			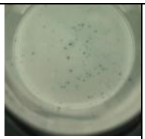				
34	0	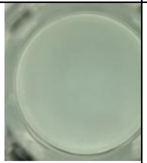	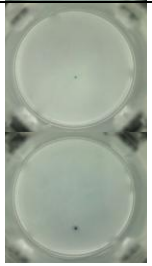	(1+1)<br>1	5	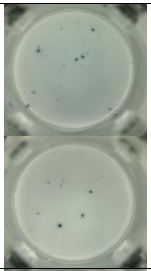	(13+9)<br>11	55	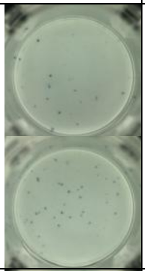	(38+14)<br>26	130	306	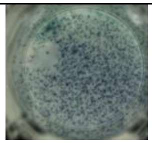
35	0	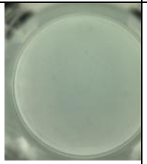	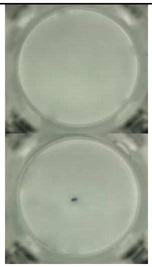	(0+2)<br>1	5	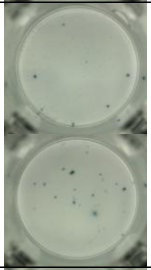	(15+13)<br>14	70	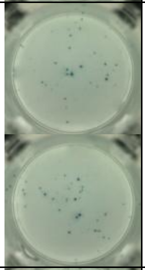	(32+28)<br>30	150	307	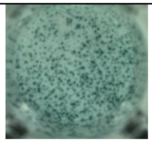
36	0	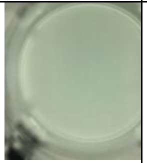	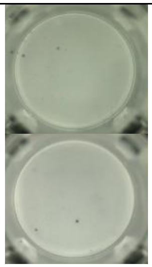	(2+2)<br>2	10	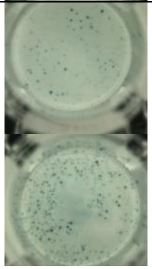	(100+94<br>)<br>97	486	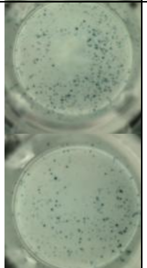	(124+164)<br>144	720	289	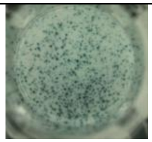
37	0	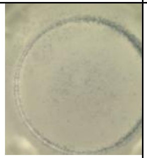	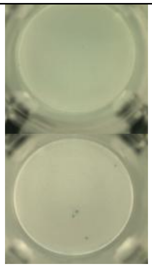	(0+4)<br>2	10	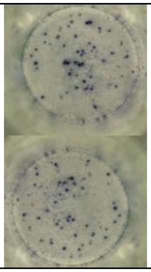	(69+65)<br>67	335	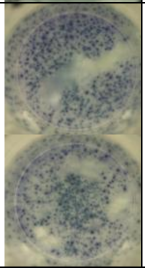	(190+194)<br>192	960	321	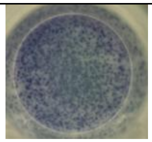

38	0	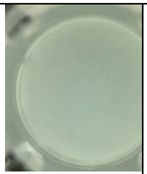	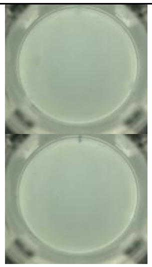	(0+0)<br>0	0	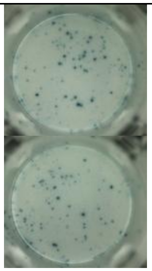	(76+68)<br>72	370	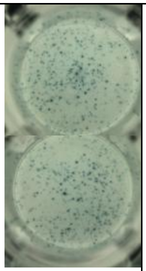	(237+223)<br>230	1150	310	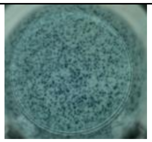
39	0	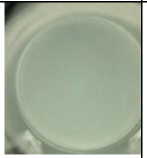	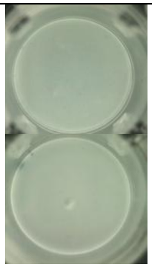	(0+1)<br>0,5	2,5	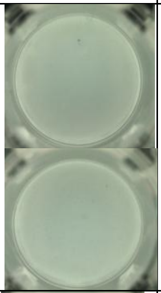	(1+1)<br>1	5	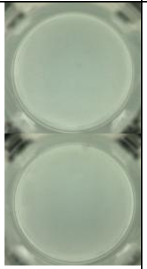	(0+0=<br>0	0	297	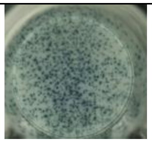
40	0	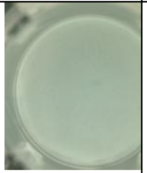	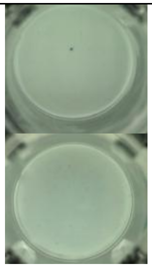	(0+1)<br>0,5	2,5	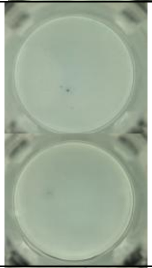	(3+3)<br>83	15	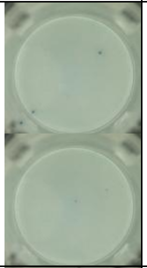	(2+2)<br>2	10	304	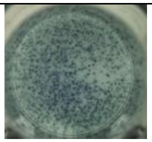
41	5	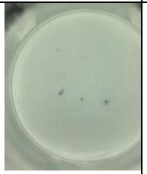	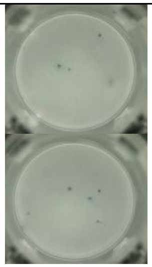	(6+8)<br>2	10	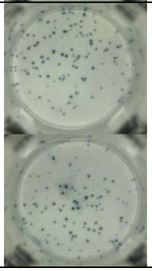	(93+90)<br>86,5	432,5	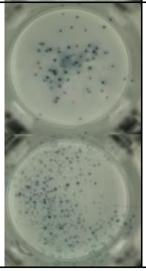	(201+69)<br>130	650	317	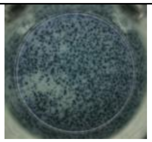

42	0	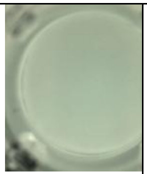	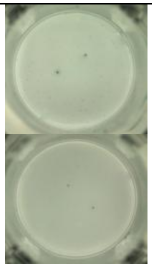	(2+2)<br>2	10	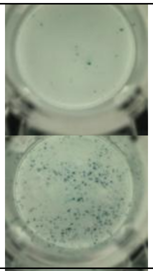	(105+87<br>)<br>96	480	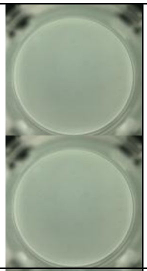	(0+0)<br>0	0	282	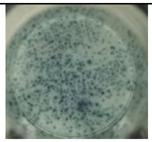
43	0	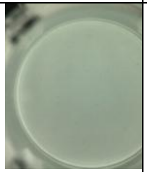	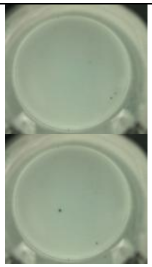	(2+2)<br>2	10	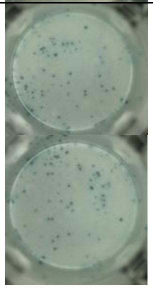	(63+55)<br>59	295	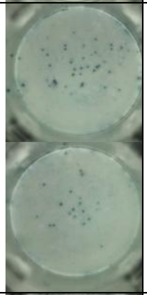	(23+43)<br>38	190	279	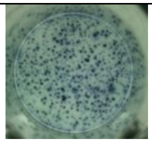
44	0	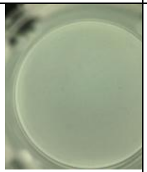	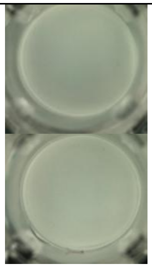	(0+0)<br>0	0	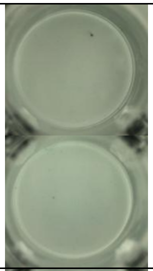	(1+1)<br>1	5	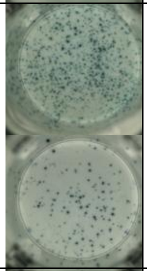	(133+379)<br>256	1280	289	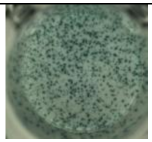
45	0	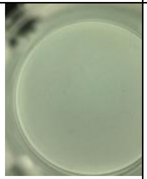	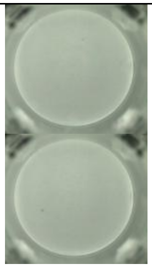	(1+1)<br>1	5	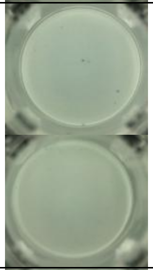	(4+6)<br>5	25	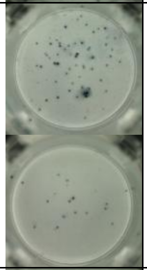	(18+58)<br>38	190	298	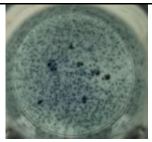
46	0	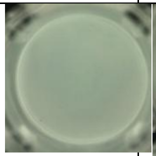	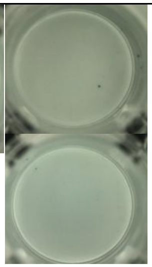	(1+1)<br>1	5	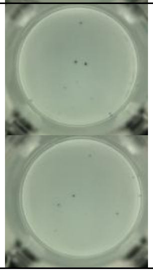	(5+7)<br>6	30	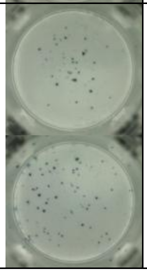	(63+39)<br>56	280	301	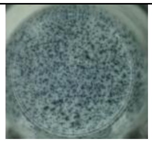

47	0	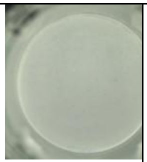	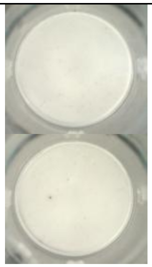	1+1)<br>1	5	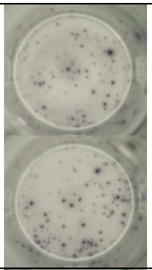	(96+88)<br>92	477,5	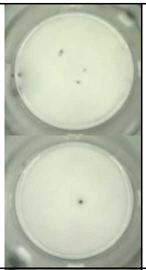	(1+5)<br>3	15	304	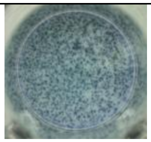
48	0	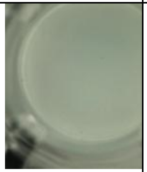	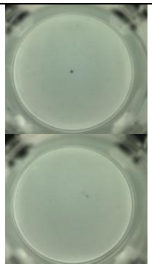	(1+1)<br>1	5	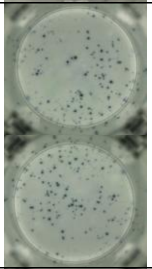	(99+105<br>)<br>102	510	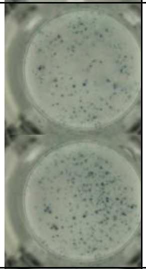	(201+133)<br>167	835	300	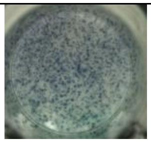
49	0	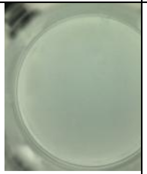	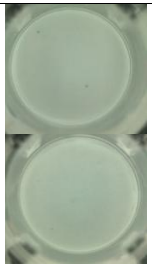	(1+2)<br>1,5	7,5	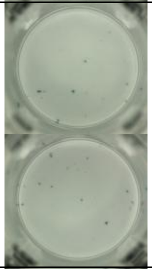	(11+7)<br>9	45	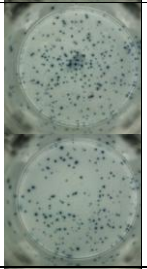	(159+209)<br>184	920	287	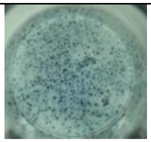
50	0	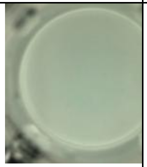	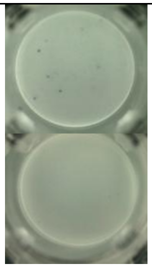	(0+4)<br>2	10	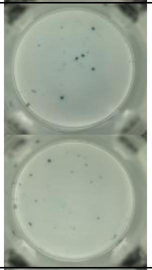	(18+13)<br>15,5	77,5	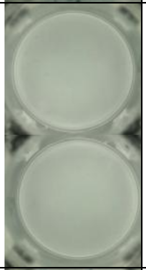	(0+0)<br>0	0	288	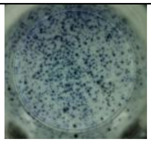
51	0	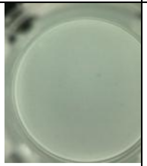	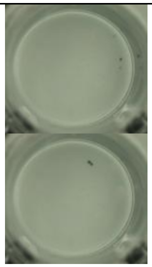	(2+2)<br>2	10	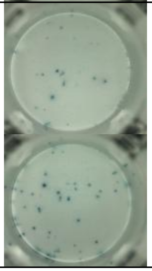	(21+27)<br>24	120	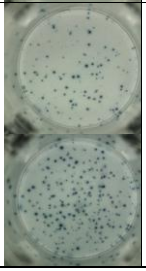	(207+85)<br>146	730	297	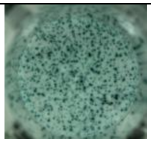

52	0	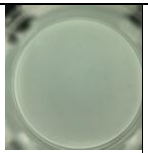	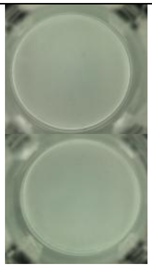	(0+0)<br>0	0	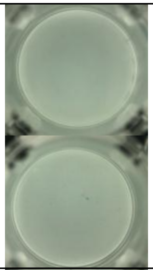	(1+0)<br>0,5	2,5	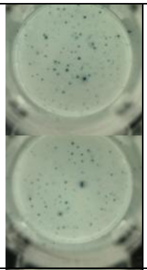	(80+76)<br>78	390	321	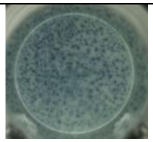
53	2	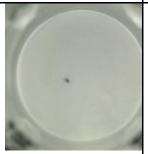	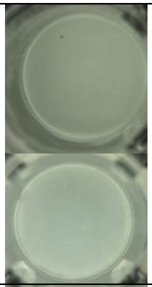	(3+2)<br>0,5	2,5	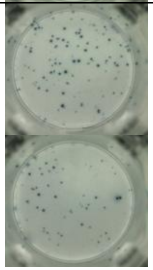	(69+65)<br>66	330	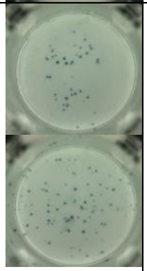	(78+44)<br>59	295	248	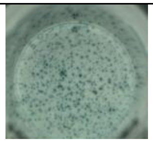
54	0	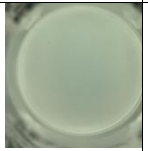	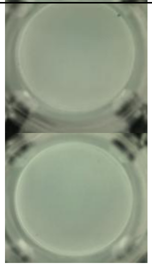	(0+0)<br>0	0	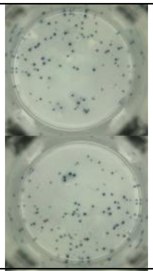	(81+79)<br>80	400	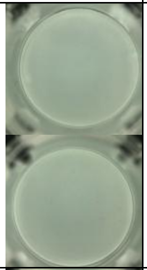	(0+0)<br>0	0	231	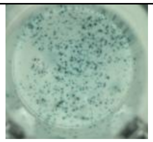
55	0	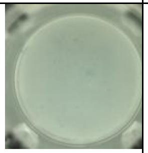	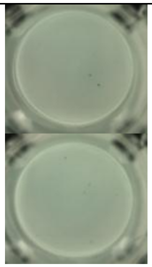	(2+2)<br>2	10	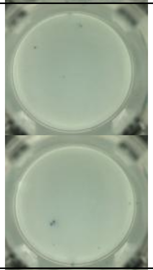	(4+2)<br>3	15	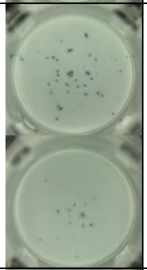	(13+43)<br>28	140	287	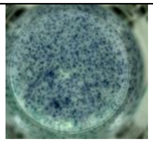
56	0	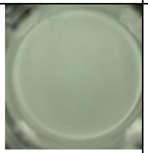	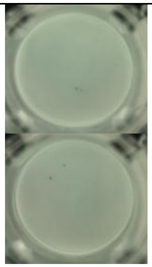	(2+2)<br>2	10	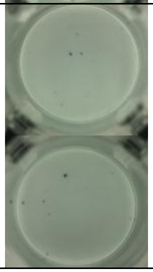	(4+5)<br>4,5	22,5	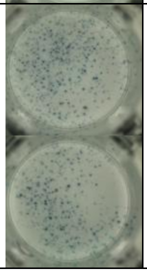	(201+218)<br>209,5	1047,5	275	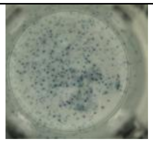

57	0	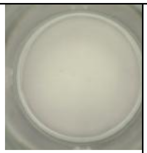	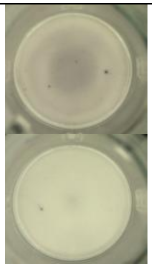	(2+2)<br>2	10	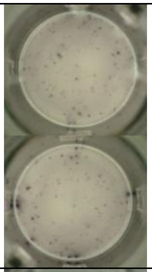	(61+50)<br>55,5	277,5	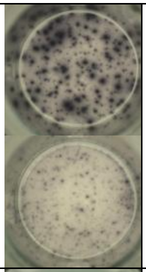	(260+224)<br>242	1210	249	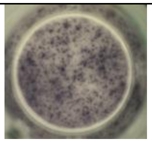
58	0	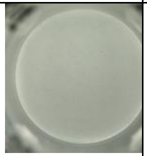	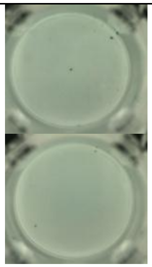	(2+2)<br>2	10	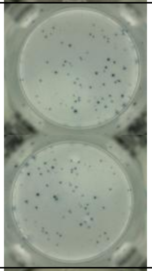	(63+61)<br>62	310	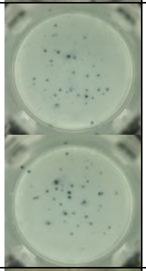	(32+42)<br>37	185	197	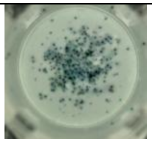
59	0	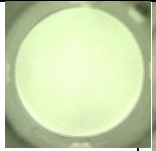	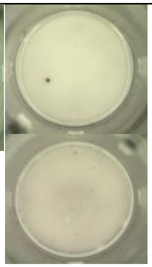	(2+2)<br>2	10	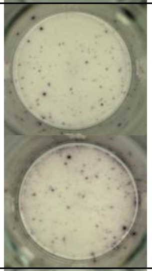	(76+78)<br>77	385	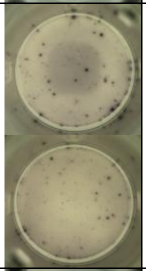	(32+46)<br>39	195	224	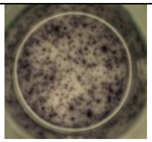
60	0	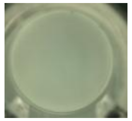	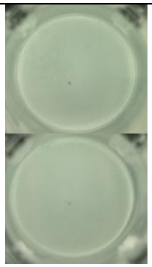	(0+2)<br>1	5	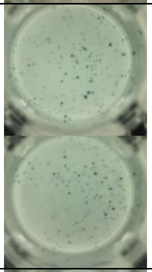	(101+97<br>)<br>99	495	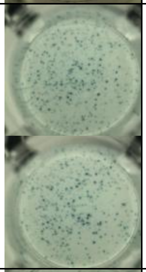	(232+240)<br>236	1180	302	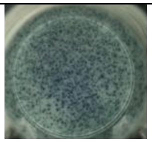
61	0	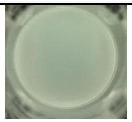	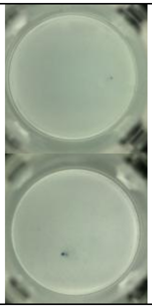	(2+1)<br>1,5	7,5	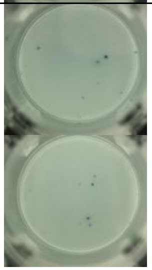	(8+8)<br>8	40	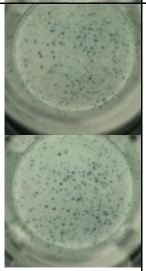	(223+175)<br>199	995	300	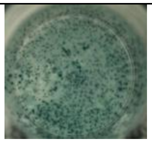

62	0	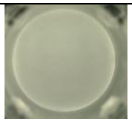	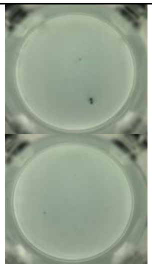	(2+2)<br>2	10	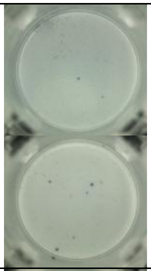	(11+13)<br>12	60	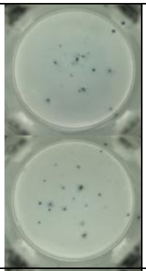	(16+20)<br>18	90	197	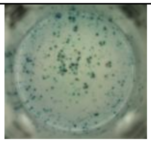
63	0	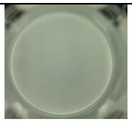	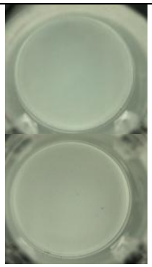	(0+1)<br>0,5	2,5	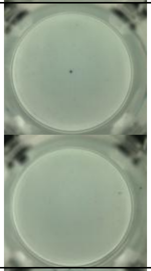	(1+1)<br>1	5	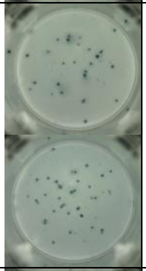	(28+42)<br>35	175	277	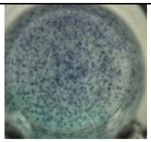
64	0	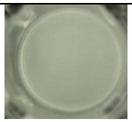	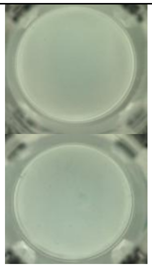	(0+0)<br>0	0	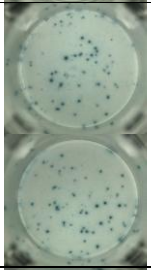	(63+57)<br>60	300	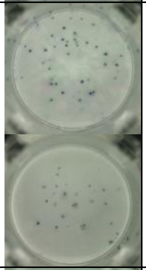	(28+40)<br>34	170	265	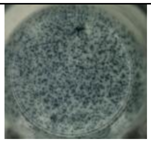
65	0	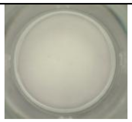	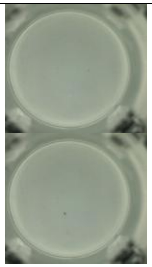	(0+1)<br>1	5	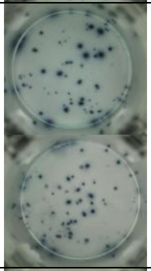	(55+57)<br>56	280	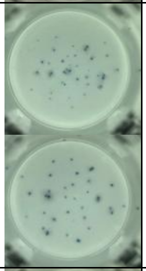	(35+41)<br>38	190	301	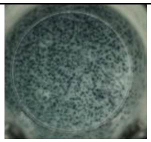
66	0	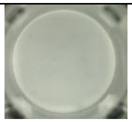	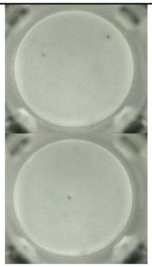	(2+1)<br>1,5	7,5	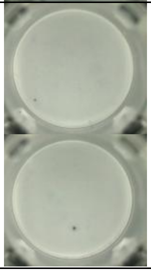	(0+1)<br>1	5	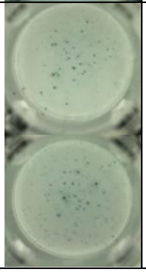	(55+34)<br>44,5	222,5	283	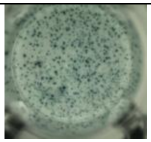

67	0	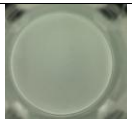	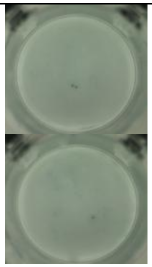	(1+2)<br>1,5	7,5	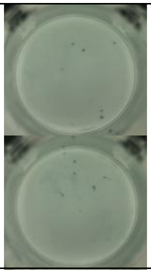	(6+6)<br>6	30	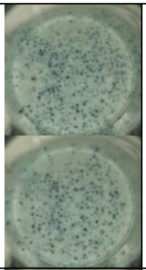	(240+178)<br>209	1045	265	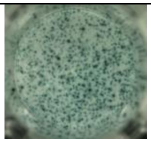
68	0	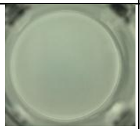	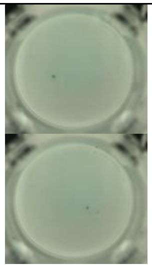	(0+2)<br>2	10	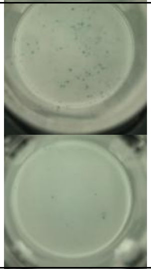	(30+12)<br>21	105	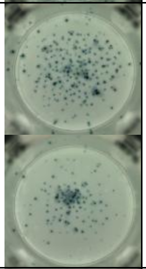	(121+245)<br>183	915	317	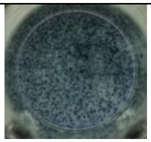
69	0	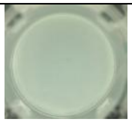	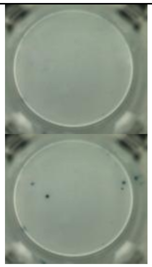	(2+2)<br>2	10	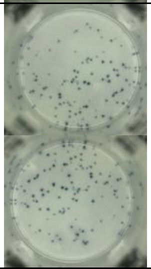	(93+91)<br>92	460	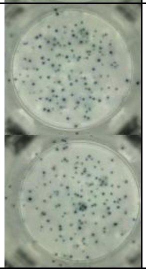	(128+140)<br>134	670	302	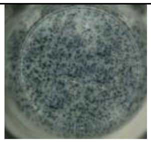
70	0	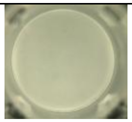	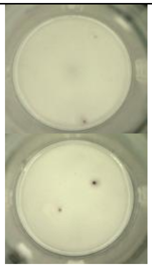	(2+2)<br>2	10	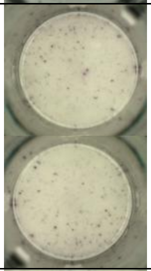	(69+69)<br>69	345	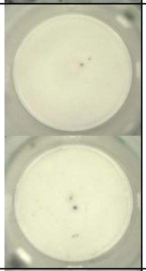	(4+2)<br>3	15	198	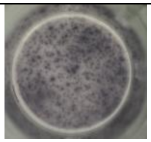
71	0	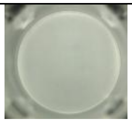	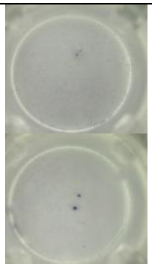	(2+0)<br>2	10	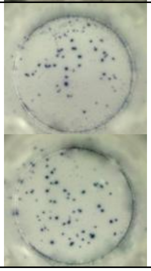	(69+59)<br>64	320	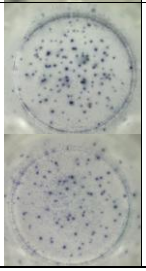	(92+140)<br>116	580	304	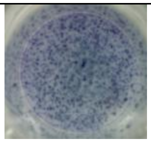

72	0	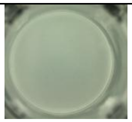	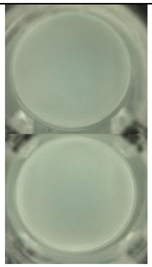	(0+0)<br>0	0	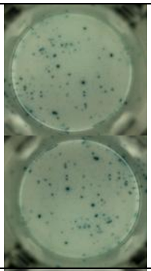	(66+66)<br>66	330	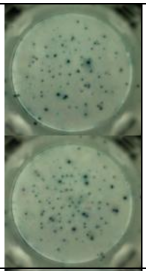	(98+88)<br>93	465	315	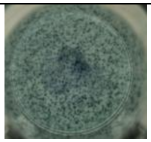
73	0	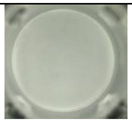	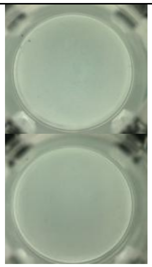	(0+1)<br>0,5	2,5	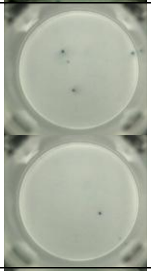	(3+5)<br>4	20	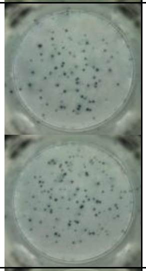	(119+82)<br>100,5	502,5	321	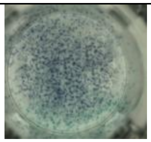
74	0	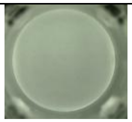	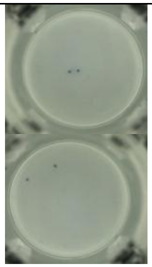	(2+2)<br>2	10	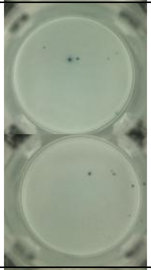	(4+6)<br>5	25	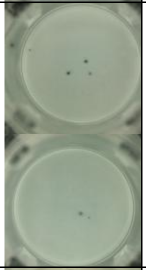	(2+4)<br>3	15	294	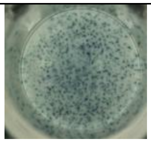
75	0	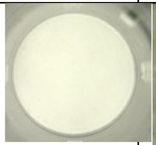	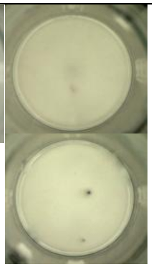	(0+2)<br>1	5	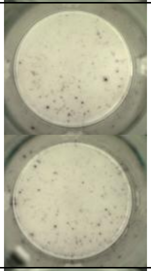	(85+74)<br>79,5	397,5	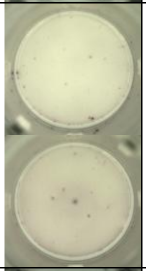	(11+13)<br>12	60	195	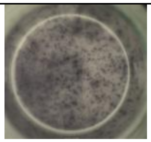
76	0	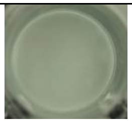	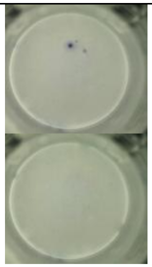	(1+0)<br>1,5	7,5	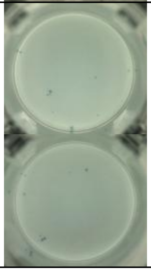	(6+7)<br>6,5	32,5	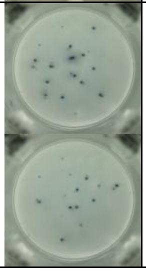	(21+31)<br>26	130	239	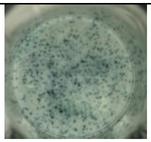

77	0	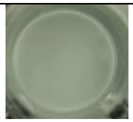	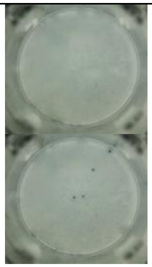	(1+3)<br>2	10	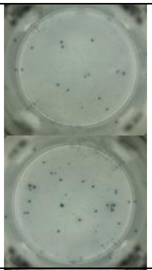	(27+23)<br>25	125	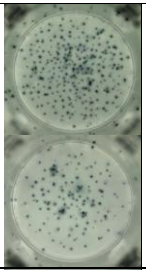	(135+277)<br>206	1030	298	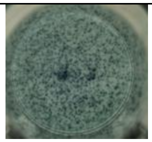
78	0	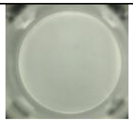	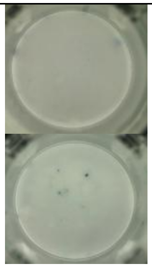	(2+2)<br>2	10	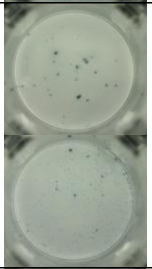	(29+27)<br>28	140	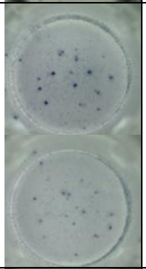	(21+23)<br>22	110	312	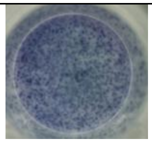
79	2	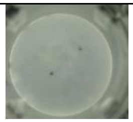	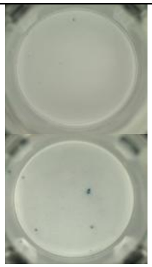	(5+3)<br>2	10	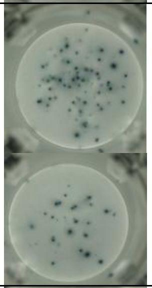	(54+60)<br>55	275	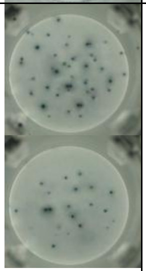	(21+45)<br>31	155	324	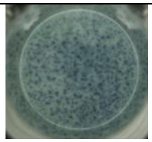
80	0	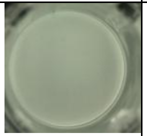	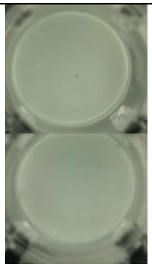	(0+1)<br>0,5	2,5	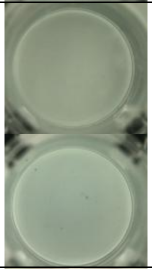	(5+7)<br>6	30	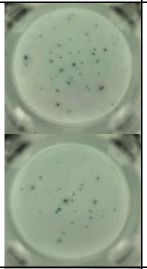	(30+40)<br>35	175	315	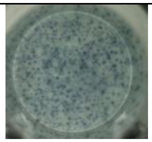
81	0	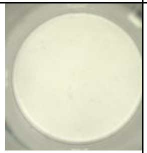	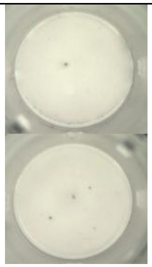	(3+1)<br>2	10	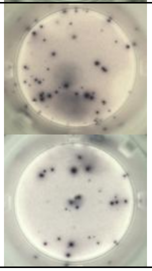	(62+68)<br>65	325	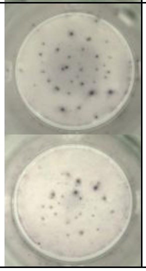	(30+46)<br>38	190	265	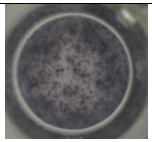

82	1	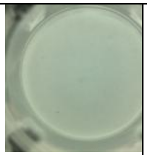	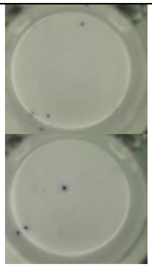	(3+3)<br>2	10	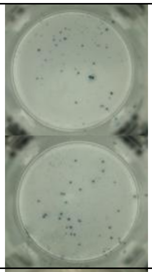	(43+41)<br>42	210	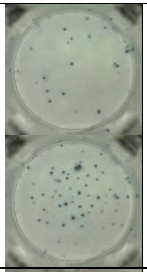	(63+23)<br>42	210	387	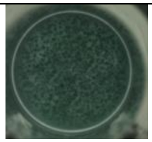
83	0	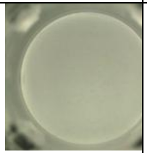	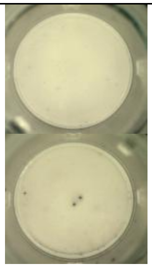	(2+1)<br>1,5	7,5	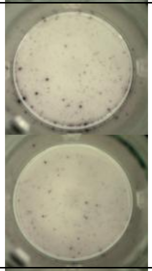	(43+61)<br>52	260	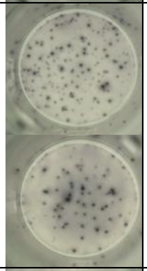	(101+149)<br>125	625	221	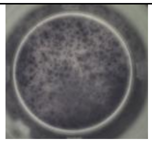
84	0	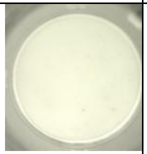	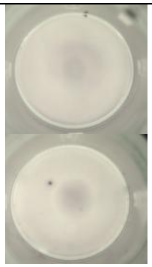	(1+0)<br>0,5	2,5	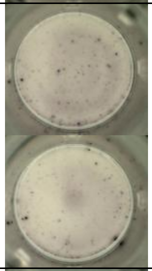	(51+55)<br>53	265	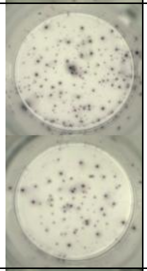	(68+150)<br>109	545	236	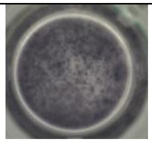
85	0	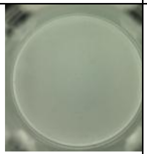	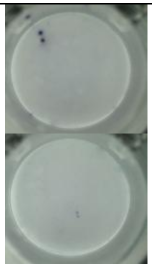	(2+2)<br>2	10	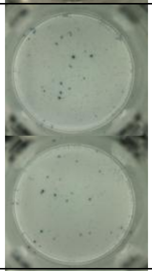	(26+30)<br>+28	140	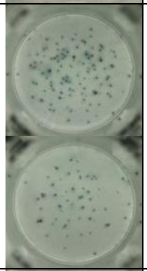	(58+120)<br>89	445	285	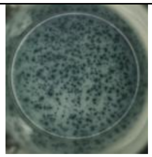
86	0	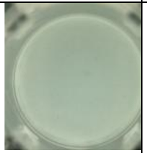	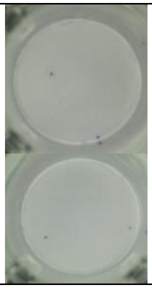	(0+4)<br>2	10	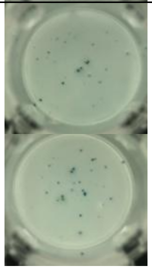	(28+23)<br>25,5	127,5	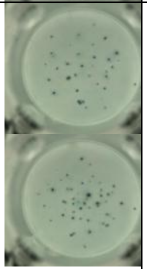	(51+37)<br>44	220	273	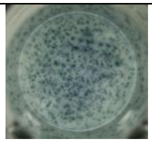

87	0	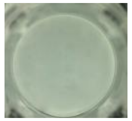	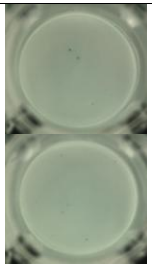	(2+2)<br>2	10	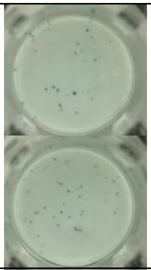	(28+32)<br>30	150	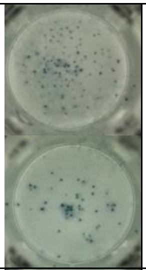	(48+86)<br>67	335	300	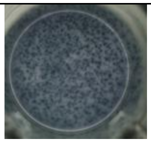
88	0	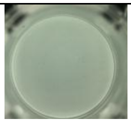	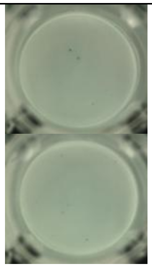	(1+3)<br>2	10	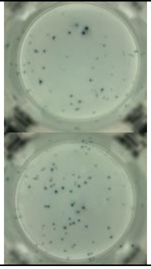	(63+29)<br>51	255	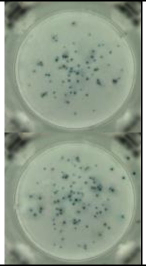	(99+79)<br>89	445	285	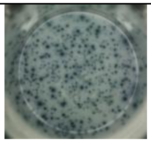

ELISpot images and spot number for ACTIVE TB PATIENTS (N=73)

N	Medium (negative control, subtracted) spot/ 0.2 x 10^6 cell	Medium Pic spot/ 0.2 x 10^6 cell	Ala-DH pic spot/ 0.2 x 10^6 cell	Ala-DH spot/ 0.2 x 10^6 cell	Ala-DH spot/ 10^6 cell	ESAT-6 pic spot/ 0.2 x 10^6 cell	ESAT-6 spot/ 0.2 x 10^6 cell	ESAT-6 spot/ 10^6 cell	CFP-10 pic spot/ 0.2 x 10^6 cell	CFP-10 spot/ 0.2 x 10^6 cell	CFP-10 spot/ 10^6 cell	PHA (positive control) spot/ 0.2 x 10^6 cell	PHA (positive control) spot/ 0.2 x 10^6 cell
1	0	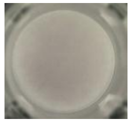	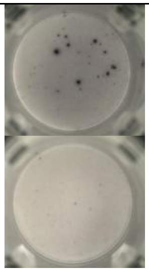	(35+15)<br>25	125	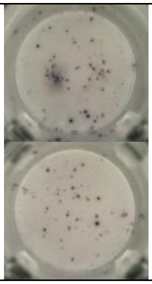	(69+69)<br>69	345	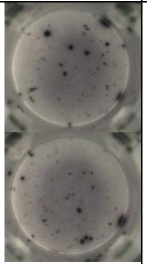	(76+79)<br>77,5	387,5	The number of spots is over the maximum number of detectable spots.	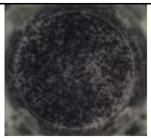

2	1	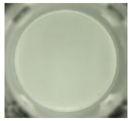	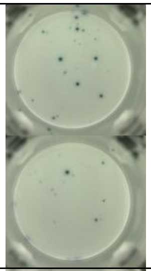	(18+12<br>)<br>14	70	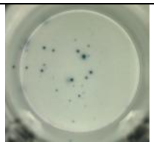	(24)<br>24	120	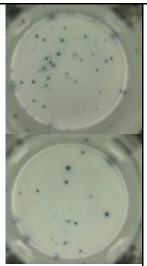	(15+35)<br>24	120	275	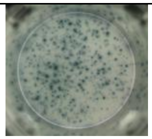
3	2	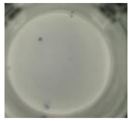	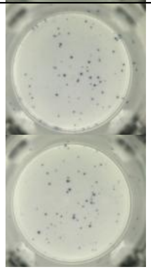	(59+57<br>)<br>56	280	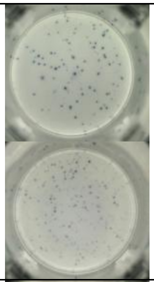	(87+105<br>)<br>94	470	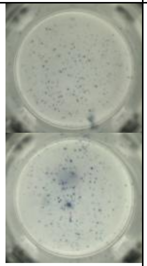	(118+134)<br>124	620	254	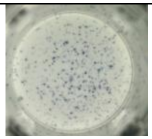
4	3	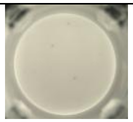	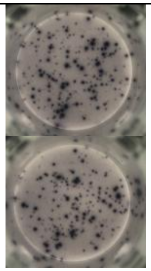	(172+1<br>66)<br>166	830	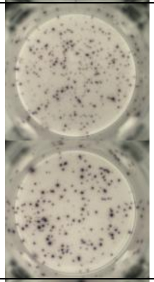	(162+20<br>0)<br>178	890,5	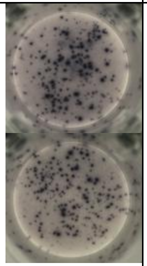	(188+208)<br>195	975	271	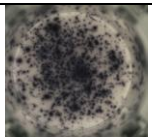
5	1	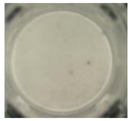	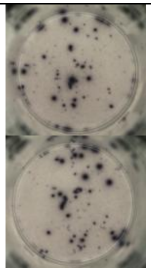	(72+74<br>)<br>72	360	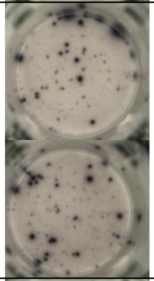	(83+76)<br>78,5	392,5	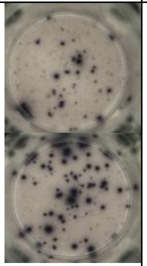	(102+92)<br>96	480	378	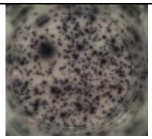
6	0	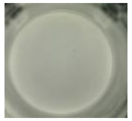	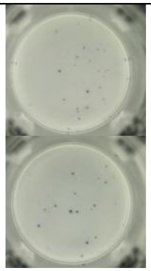	(17+19<br>)<br>18	90	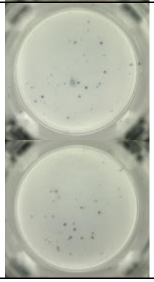	(29+27)<br>28	140	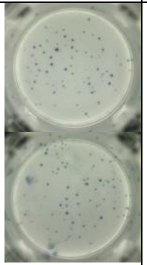	(66+64)<br>65	325	263	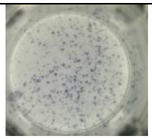

7	1	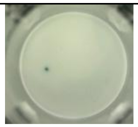	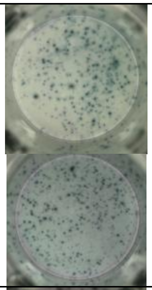	(219+231)224	1120	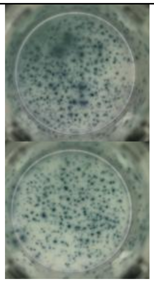	(301+289)294	1470	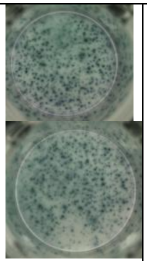	(332+312)<br>321	1605	279	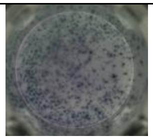
8	2	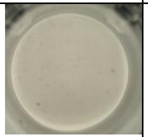	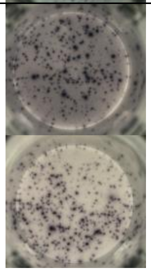	(272+252)260	1300	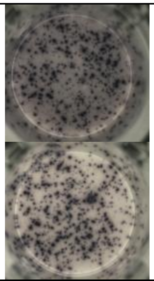	(322+318)318	1590,5	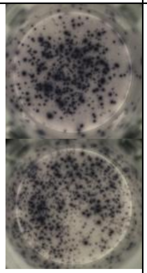	(339+333)<br>334	1670	275	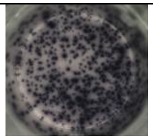
9	1	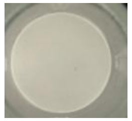	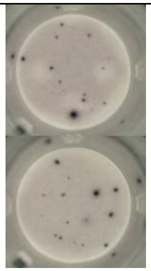	(16+16)<br>15	75	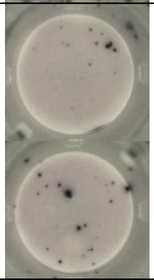	(24+17)<br>19,5	97	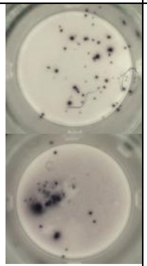	(64+50)<br>56	280	The number of spots is over the maximum number of detectable spots.	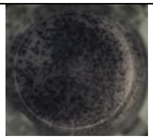
10	2	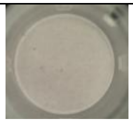	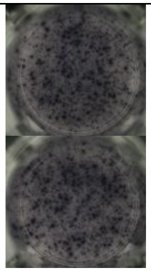	(240+256)<br>246	1230	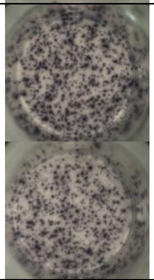	(256+268)<br>260	1300	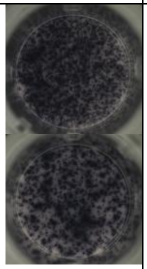	(331+309)<br>318	1590	The number of spots is over the maximum number of detectable spots.	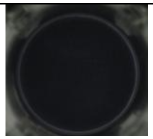
11	0	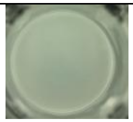	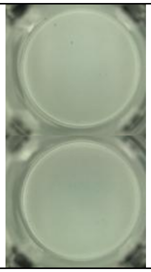	(0+2)<br>1	5	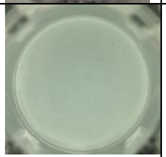	(3+1)<br>2	10	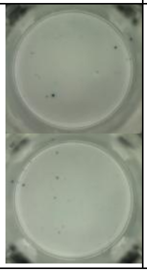	(6+6)<br>6	30	294	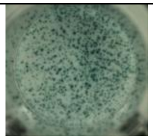

						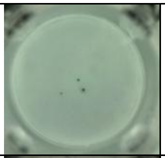							
12	0	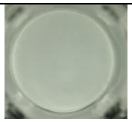	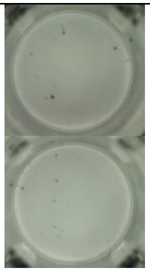	(7+7)<br>7	35	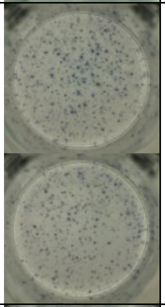	(253+22<br>1)<br>237	1185	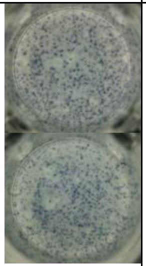	(328+308)<br>318	1590	306	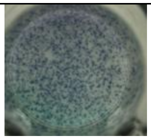
13	0	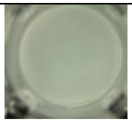	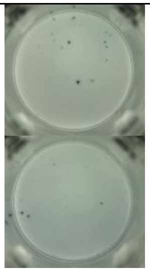	(8+12)<br>10	50	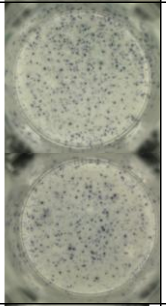	(286+30<br>8)<br>297	1485	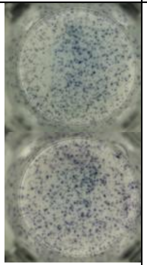	(404+400)<br>402	2010	300	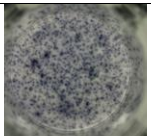
14	0	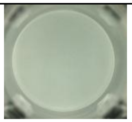	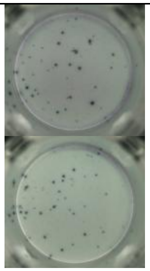	(36+36<br>)<br>36	180	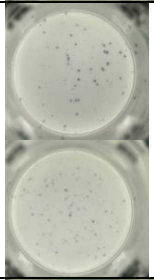	(56+65)<br>58	290	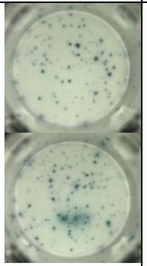	(86+98)<br>92	460	288	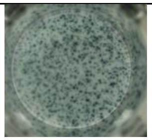
15	0	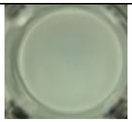	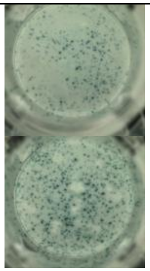	(320+1<br>90)<br>255	1275	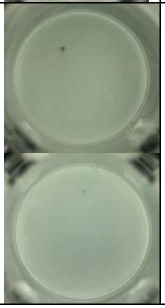	(1+1)<br>1	5	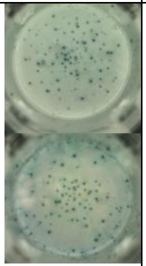	(92+72)<br>82	410	243	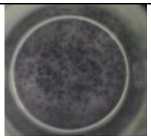

16	3	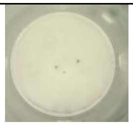	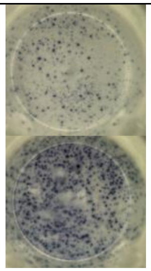	(200+320)<br>260	1300	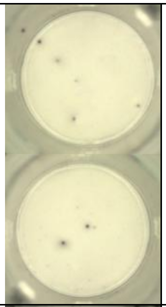	(3+5)<br>1	5	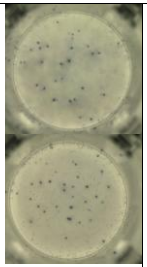	(31+35)<br>30	150	225	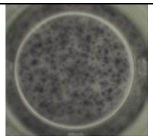
17	0	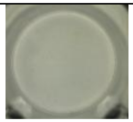	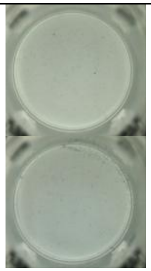	(6+4)<br>5	25	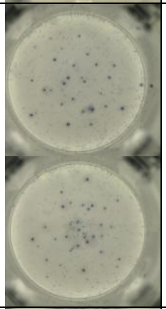	(29+35)<br>32	160	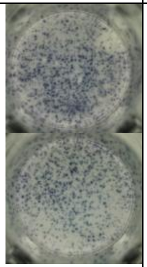	(412+434)<br>423	2115	284	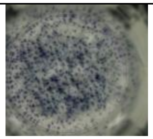
18	0	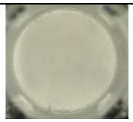	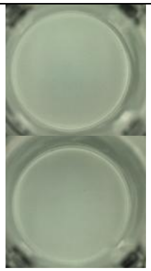	(0+0)<br>0	0	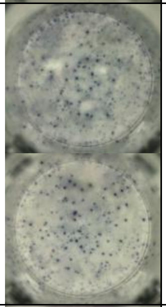	(70+66)<br>68	340	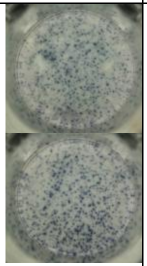	(381+353)<br>367	1835	299	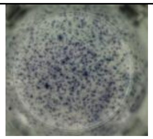
19	0	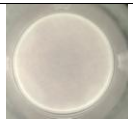	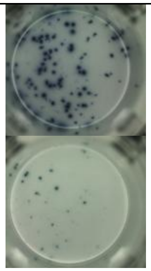	(86+20)<br>53	25	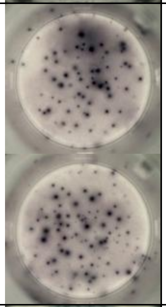	(87+71)<br>79	395	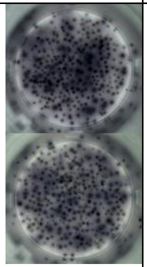	(300+338)<br>319	1595	202	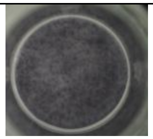
20	0	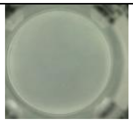	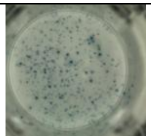	(209+231)<br>220	1100	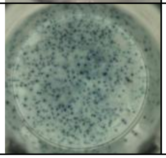	(391+383)<br>387	1935	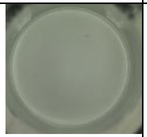	(1+1)<br>1	5	286	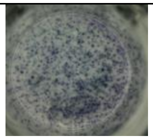

			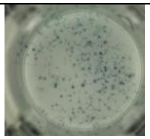			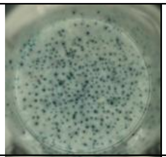			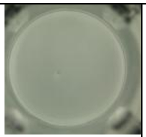				
21	0	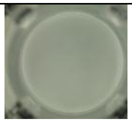	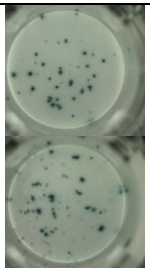	(40+60<br>)<br>50	250	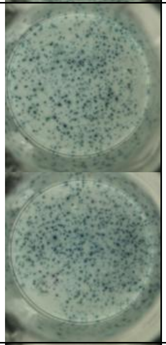	(395+36<br>9)<br>382	1910	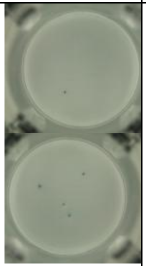	(1+1)<br>1	5	213	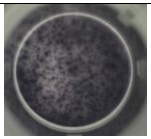
22	0	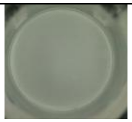	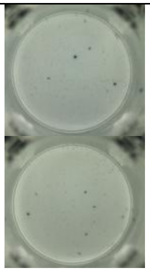	(12+14<br>)<br>13	65	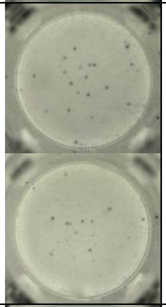	(12+19)<br>15,5	77,5	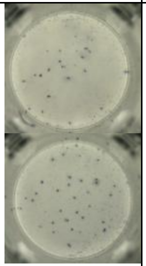	(38+28)<br>33	165	304	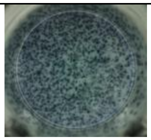
23	0	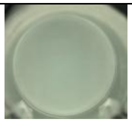	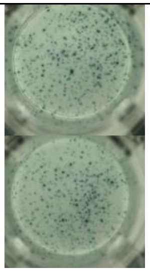	(264+3<br>02)<br>283	1415	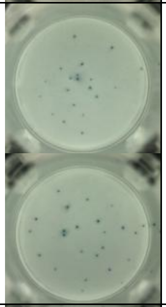	(20+24)<br>22	110	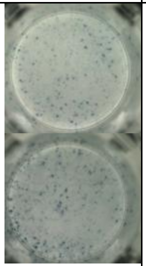	(78+86)<br>82	410	316	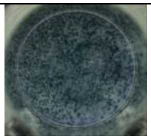
24	0	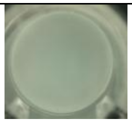	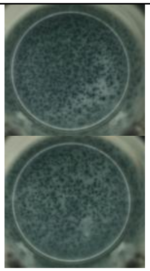	(266+2<br>77)<br>271,5	1357,5	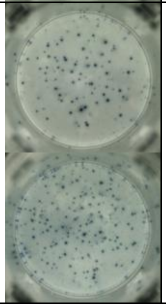	(67+53)<br>60	300	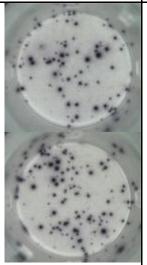	(100+71)<br>85,5	427,5	327	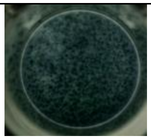

25	0	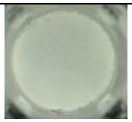	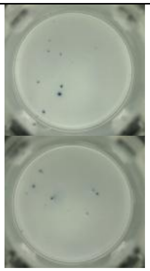	(7+7)<br>7	35	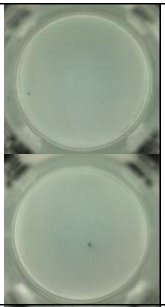	(1+1)<br>1	5	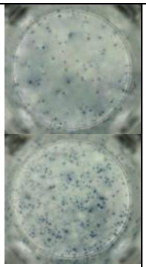	(109+71)<br>90	450	288	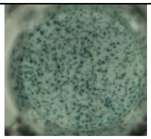
26	0	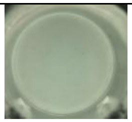	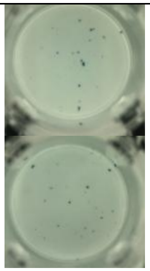	(17+19<br>)<br>18	90	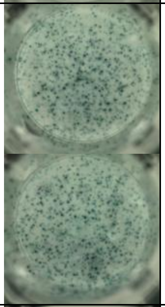	(353+36<br>1)<br>357	1785	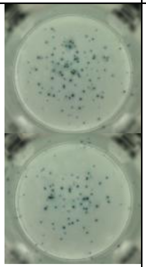	(78+110)<br>94	470	285	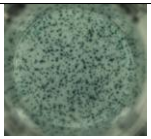
27	0	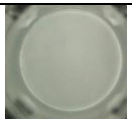	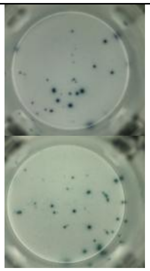	(23+25<br>)<br>24	120	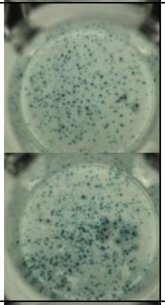	(300+28<br>2)<br>291	1455	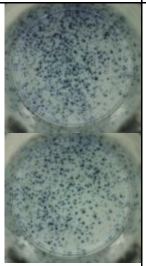	(426+434)<br>430	2150	366	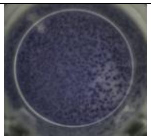
28	0	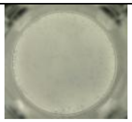	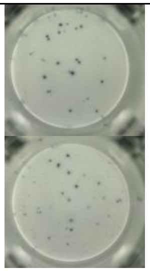	(25+29<br>)<br>27	135	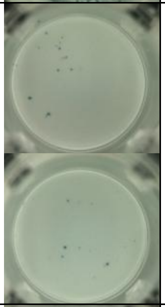	(14+10)<br>12	60	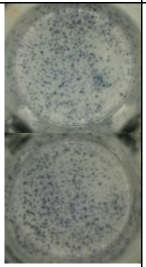	(367+358)<br>362	1810	302	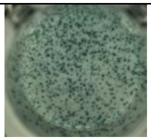
29	0	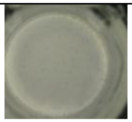	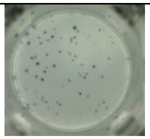	(85+63<br>)<br>74	370	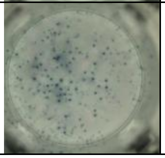	(148+98<br>)<br>123	615	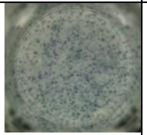	(320+329)<br>324,5	1622,5	315	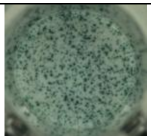

			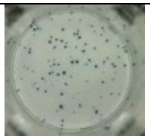			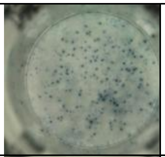			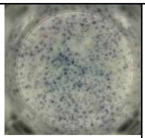				
30	0	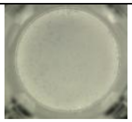	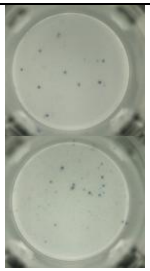	(19+13<br>)<br>16	80	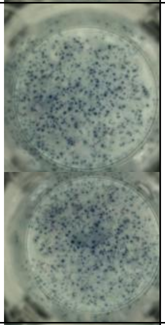	(365+36<br>1)<br>363	1815	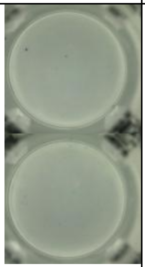	(0+2)<br>1	5	338	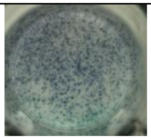
31	0	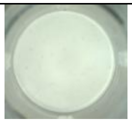	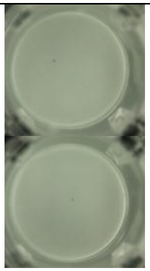	(1+1)<br>1	5	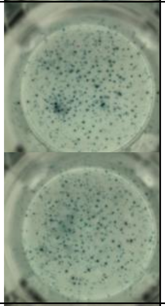	(264+30<br>6)<br>285	1425	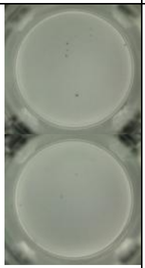	(3+4)<br>3,5	17,5	321	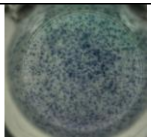
32	0	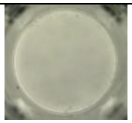	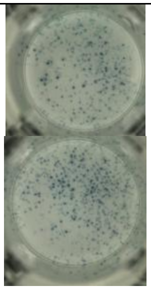	(303+2<br>15)<br>259	1295	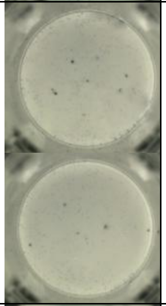	(5+7)<br>6	30	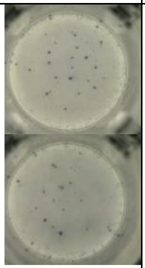	(25+27)<br>26	130	281	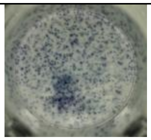
33	0	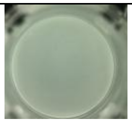	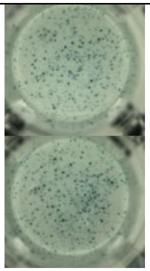	(270+2<br>90)<br>280	1400	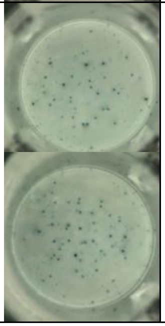	(48+38)<br>43	215	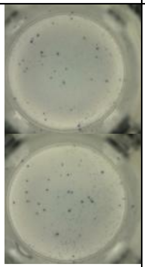	(34+40)<br>37	185	290	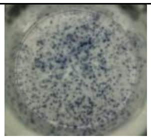

34	0	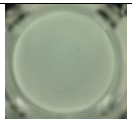	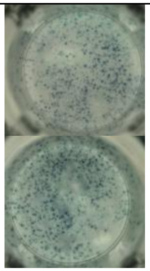	(250+2<br>14)<br>232	1160	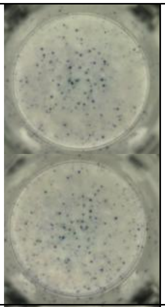	(57+48)<br>52	260	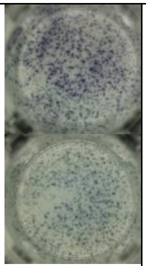	(377+423)<br>400	2000	327	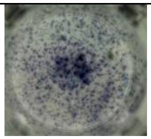
35	0	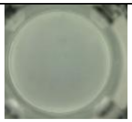	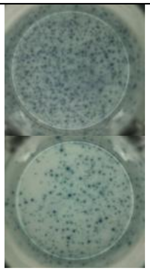	(194+3<br>04)<br>249	1245	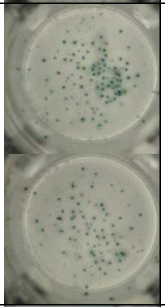	(84+98)<br>91	455	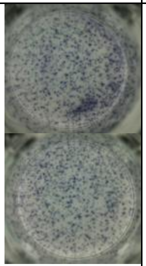	(427+415)<br>421	2105	342	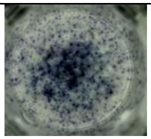
36	2	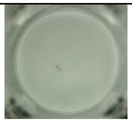	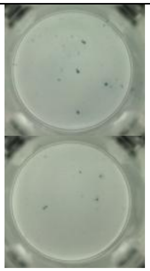	(10+6)<br>8	40	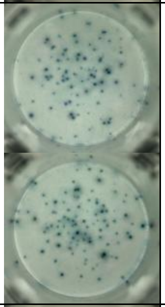	(98+90)<br>92	460	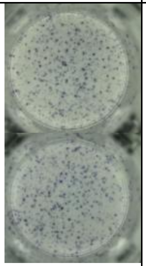	(287+303)<br>287	1435	298	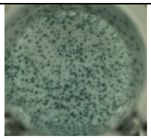
37	0	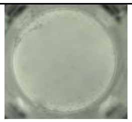	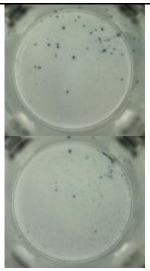	(34+38<br>)<br>36	180	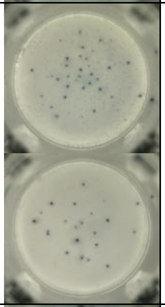	(36+22)<br>29	145	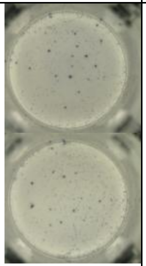	(69+61)<br>65	325	289	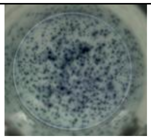
38	0	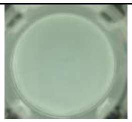	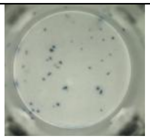	(36+52<br>)<br>44	220	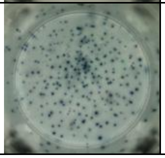	(196+24<br>6)<br>221,6	1108	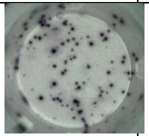	(99+87)<br>93	465	279	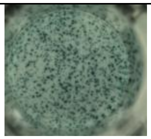

			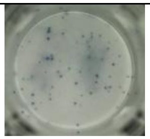			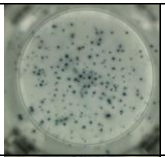			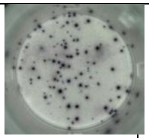				
39	4	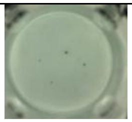	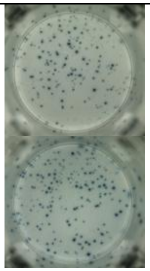	(137+159)<br>148	740	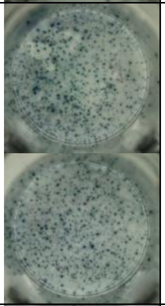	(360+350)<br>351	1755	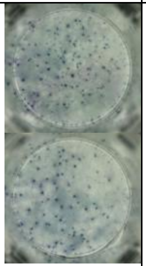	(124+80)<br>98	490	310	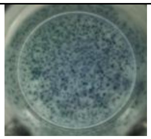
40	0	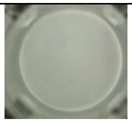	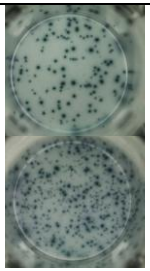	(256+109)<br>182,5	912,5	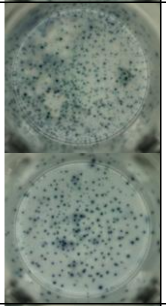	(250+316)<br>283	1415	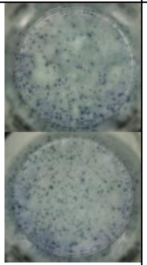	(133+109)<br>121	605	The number of spots is over the maximum number of detectable spots.	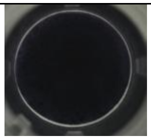
41	0	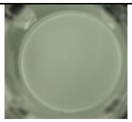	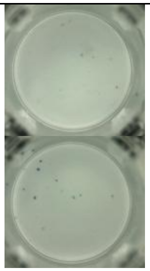	(6+10)<br>8	40	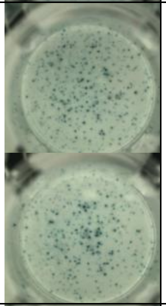	(256+246)<br>251	1255	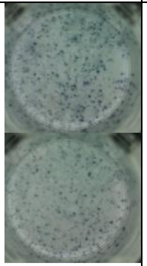	(138+122)<br>130	650	287	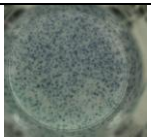
42	0	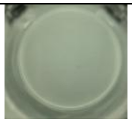	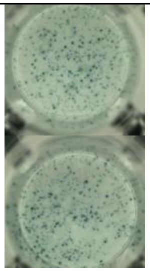	(260+262)<br>261	1305	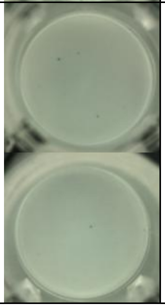	(1+3)<br>2	10	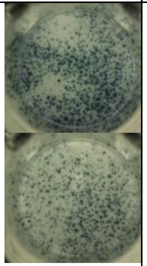	(430+424)<br>427	2135	291	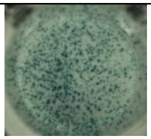

43	0	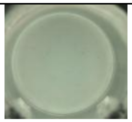	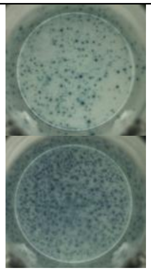	(184+251)<br>237,5	1187,5	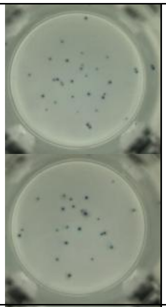	(31+23)<br>27	135	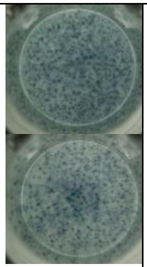	(352+400)<br>376	1880	287	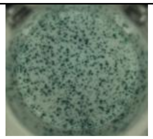
44	0	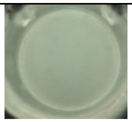	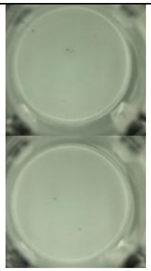	(2+4)<br>3	15	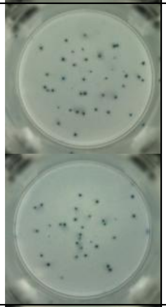	(32+38)<br>35	175	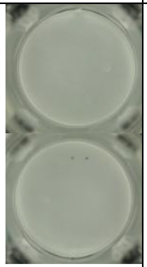	(2+0)<br>1	5	302	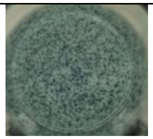
45	0	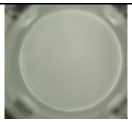	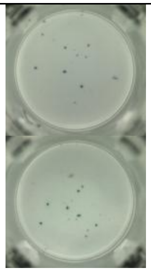	(12+12)<br>12	60	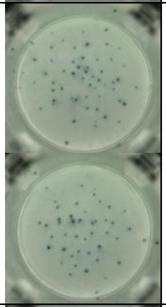	(45+55)<br>50	250	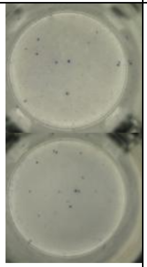	(13+11)<br>12	60	290	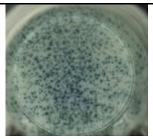
46	0	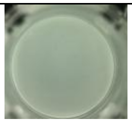	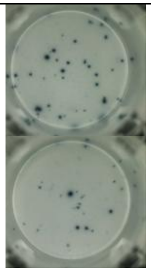	(20+26)<br>23	115	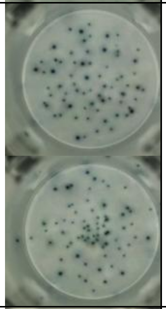	(72+72)<br>72	360	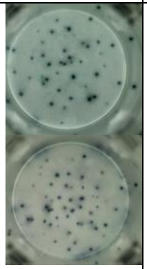	(54+42)<br>48	240	285	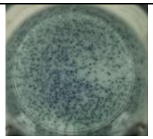
47	0	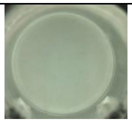	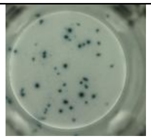	(41+49)<br>45	225	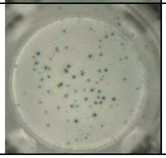	(80+86)<br>83	415	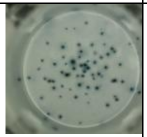	(62+70)<br>66	330	349	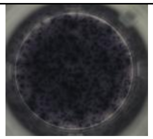

			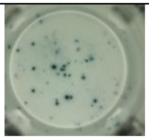			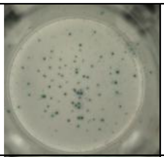			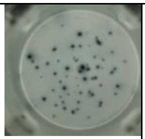				
48	0	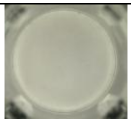	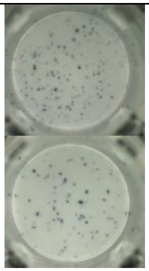	(114+74)<br>94	470	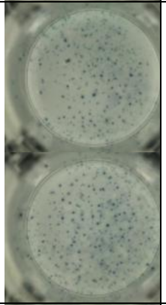	(143+151)<br>146,8	734	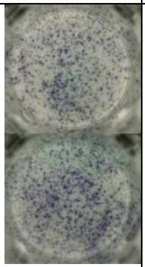	(395+427)<br>411	2055	296	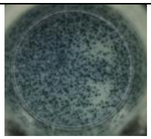
49	0	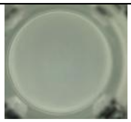	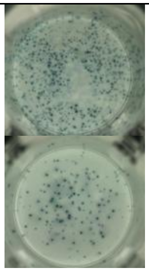	(138+268)<br>203	1015	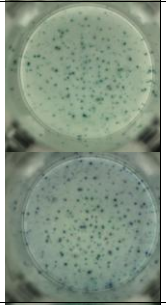	(180+184)<br>182	910	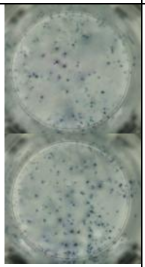	(115+67)<br>91	455	275	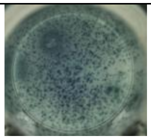
50	0	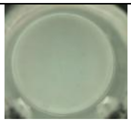	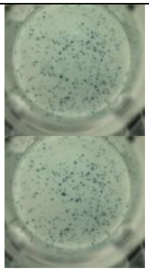	(223+223)<br>223	1115	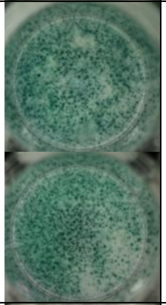	(352+328)<br>340	1700	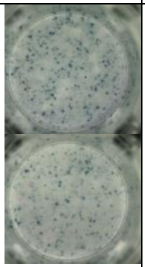	(116+102)<br>109	545	280	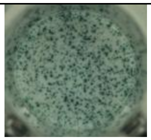
51	0	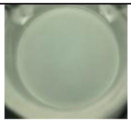	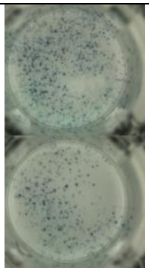	(201+319)<br>260	1300	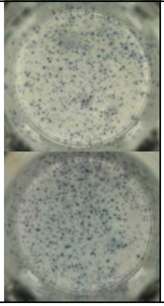	(299+301)<br>300	1500	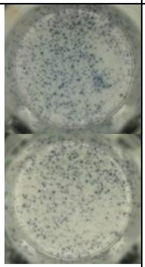	(334+356)<br>345	1725	301	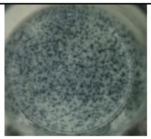

52	0	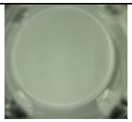	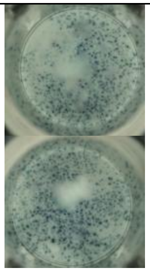	(290+192)<br>241	1205	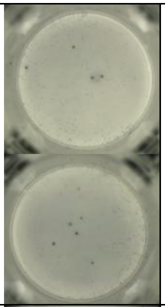	(6+4)<br>5	25	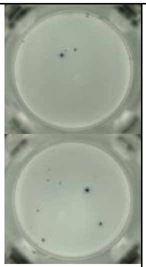	(7+3)<br>5	25	325	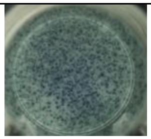
53	0	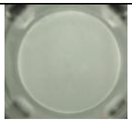	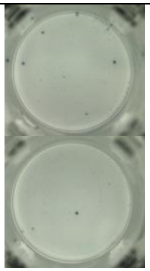	(8+4)<br>6	30	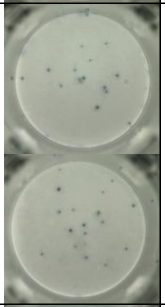	(18+11)<br>14,5	72,5	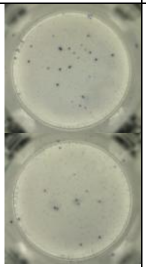	(14+26)<br>20	100	341	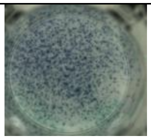
54	0	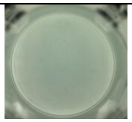	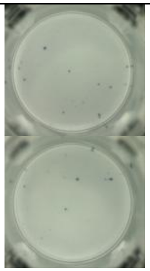	(9+9)<br>9	45	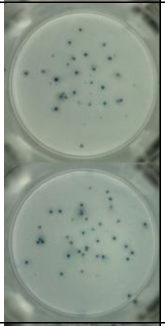	(39+31)<br>35	175	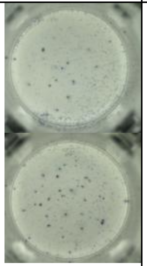	(50+58)<br>54	270	345	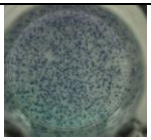
55	0	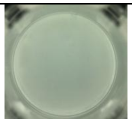	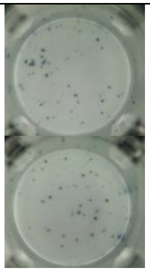	(35+43)<br>39	195	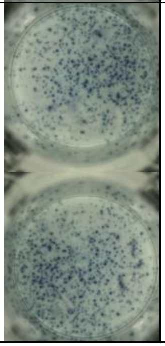	(321+321)<br>321	1605	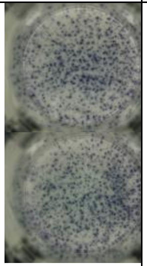	(399+380)<br>389,5	1947,5	198	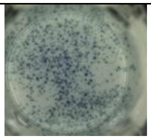

56	0	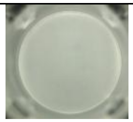	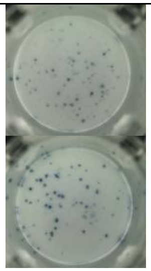	(58+56<br>)<br>57	285	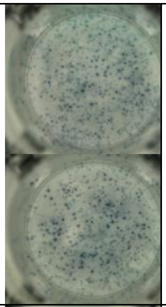	(303+30<br>1)<br>302	1510	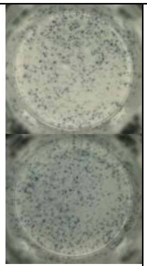	(338+322)<br>330	1650	297	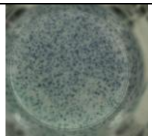
57	0	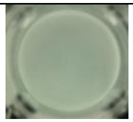	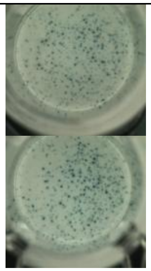	(268+2<br>42)<br>255	1275	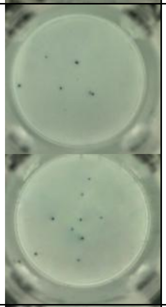	(9+7)<br>8	40	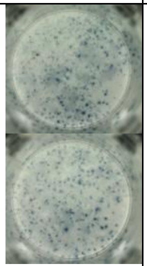	(160+140)<br>150	750	285	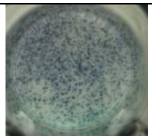
58	0	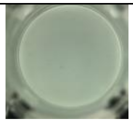	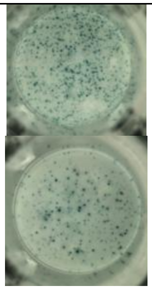	(320+1<br>86)<br>253	1265	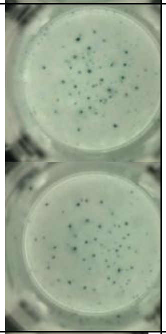	(46+38)<br>42	210	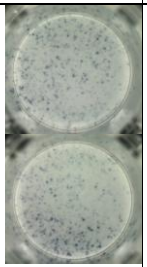	(207+195)<br>198	990	263	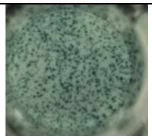
59	0	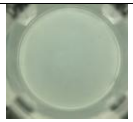	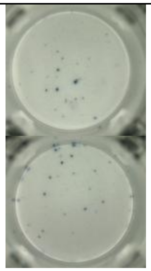	(23+15<br>)<br>19	95	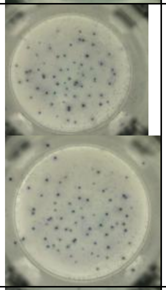	(66+64)<br>65	325	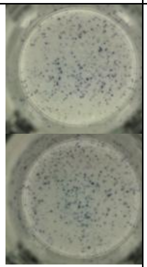	(244+242)<br>243	1215	302	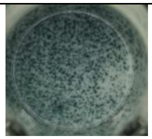
60	0	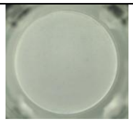	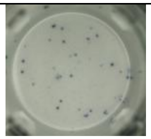	(30+32<br>)<br>31	155	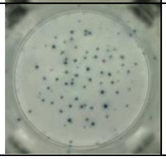	(58+64)<br>61	305	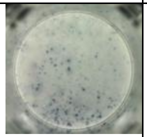	(100+76)<br>88	440	316	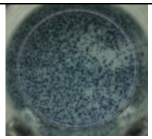

			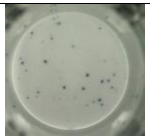			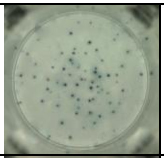			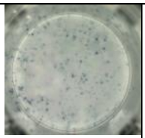				
61	0	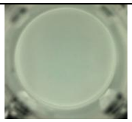	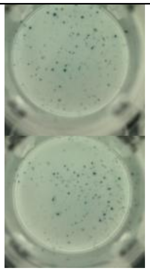	(80+97<br>)<br>88,5	442,5	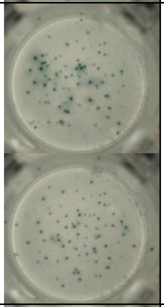	(82+88)<br>85	425	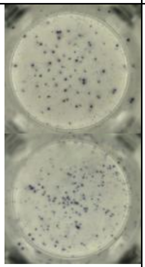	(79+77)<br>78	390	275	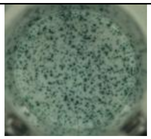
62	0	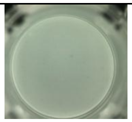	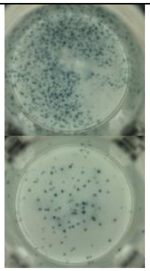	(108+3<br>28)<br>218	1090	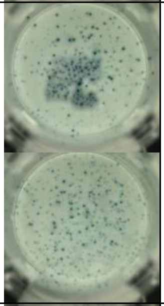	(194+21<br>0)<br>202	1010	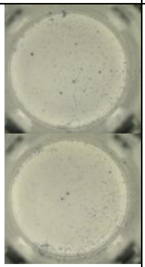	(76+68)<br>72	360	268	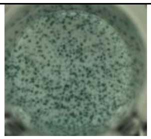
63	0	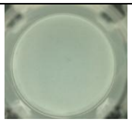	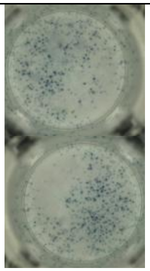	(224+2<br>48)<br>224	1120	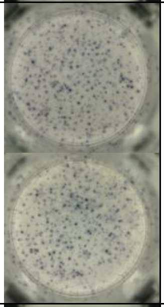	(309+30<br>5)<br>307	1535	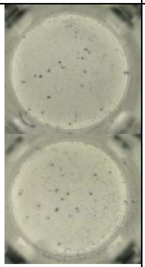	(103+79)<br>91	455	289	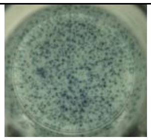
64	0	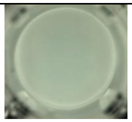	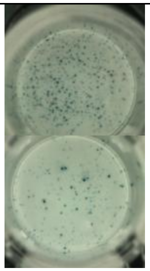	(108+2<br>12)<br>160	800	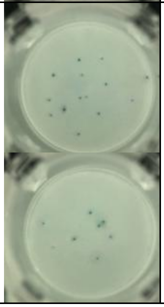	(8+14)<br>11	55	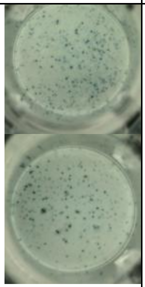	(145+163)<br>154	770	275	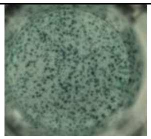

65	0	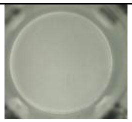	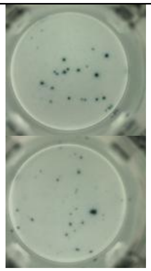	(22+22) 22	110	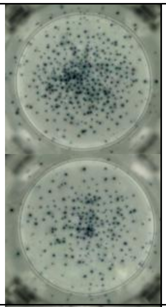	(233+345) 289	1445	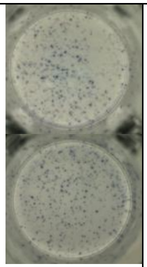	(221+203) 212	1060	273	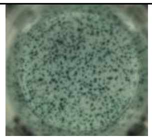
66	0	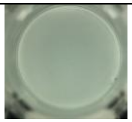	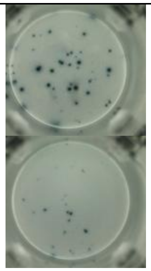	(15+37) 26	130	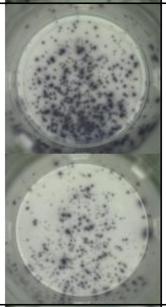	(254+286) 260	1300	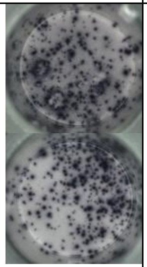	(246+256) 251	1255	289	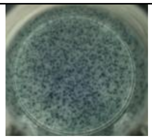
67	0	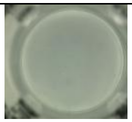	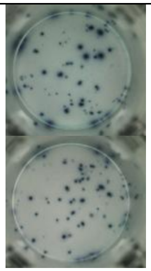	(55+55) 55	275	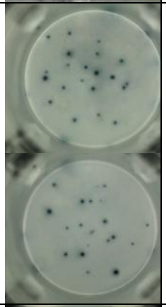	(20+21) 20,5	102,5	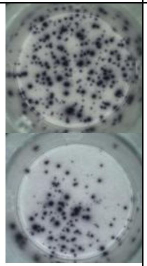	(115+215) 165	825	286	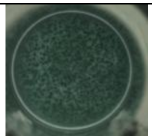
68	0	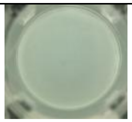	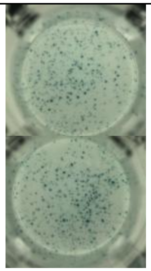	(247+261) 254	1270	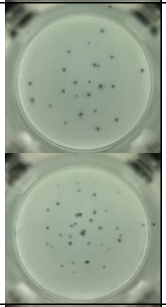	(23+33) 28	140	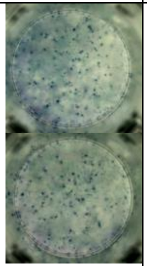	(91+96) 93,5	467,5	309	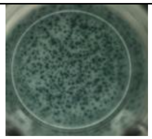
69	0	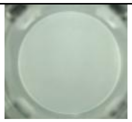	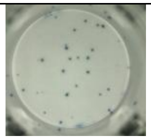	(20+26) 23	115	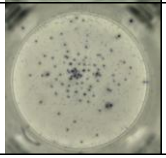	(68+88) 78	390	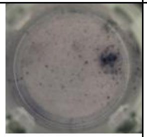	(95+73) 84	420	312	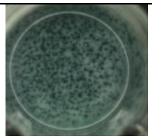

			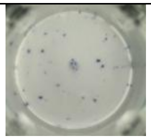			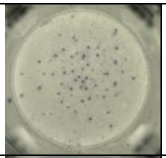			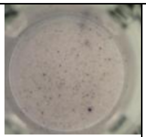				
70	0	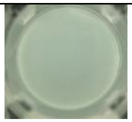	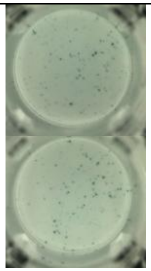	(55+65<br>)<br>60	300	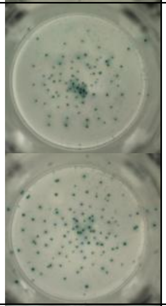	(94+90)<br>92	460	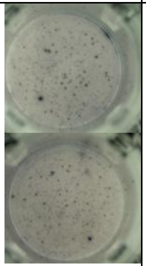	(117+147)<br>132	660	386	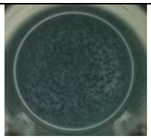
71	2	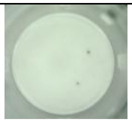	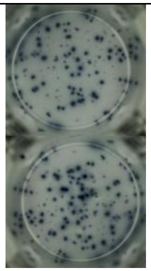	(112+9<br>2)<br>102	510	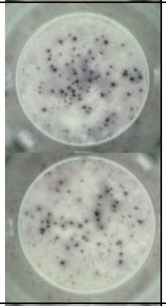	(85+65)<br>73	365	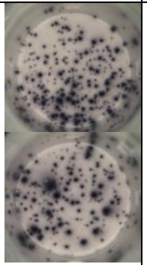	(157+199)<br>176	880	365	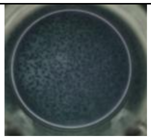
72	0	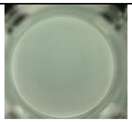	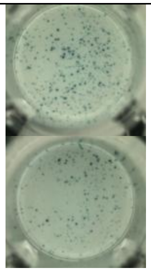	(124+2<br>32)<br>178	890	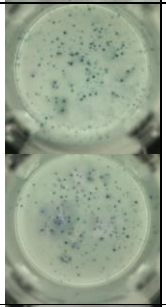	(139+95<br>)<br>119	595	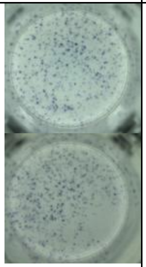	(267+285)<br>276	1380	237	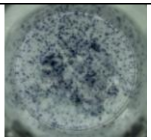
73	0	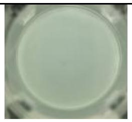	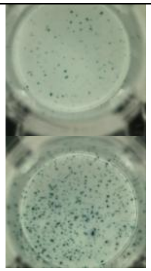	(75+30<br>5)<br>190	950	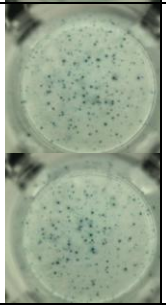	(178+14<br>0)<br>159	795	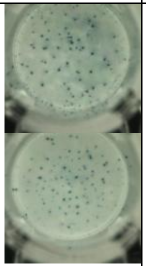	(82+70)<br>76	380	304	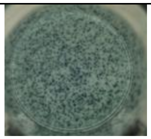
